# Serum-Based Detection of Pancreatic and Ovarian Cancer via a Nanoparticle-Enhanced Fluorescence Array and Machine Learning

**DOI:** 10.1021/acs.analchem.5c00974

**Published:** 2025-06-23

**Authors:** Violeta Morcuende-Ventura, Oscar Sánchez-Gracia, Natalia Abian-Franco, Isabel Jiménez-Pardo, Lucía Herrer, Martín Castillo-Vallés, Alexandre Lancelot, F. Javier Falcó-Martí, Sonia Hermoso-Durán, Roberto Pazo-Cid, Ángel Lanas, Adrián Velazquez-Campoy, Teresa Sierra, Olga Abian

**Affiliations:** † 82976 Instituto de Nanociencia y Materiales de Aragón (INMA), CSIC-Universidad de Zaragoza, 50009 Zaragoza, Spain; ‡ Departamento de Química Orgánica, Facultad de Ciencias, 16765 Universidad de Zaragoza, 50009 Zaragoza, Spain; § Departamento de Bioquímica y Biología Molecular y Celular, Universidad de Zaragoza, 50009 Zaragoza, Spain; ∥ Hospital Reina Sofía, Carr. Tarazona, Km. 4, 31500 Tudela, Navarra, Spain; ⊥ Institute of Biocomputation and Physics of Complex Systems (BIFI), Universidad de Zaragoza, Zaragoza 50018, Spain; # Instituto de Investigación Sanitaria Aragón (IIS Aragón), 50009 Zaragoza, Spain; 7 Hospital Universitario Miguel Servet (HUMS), Paseo Isabel la Católica, 1-3, 50009 Zaragoza, Spain; 8 Hospital Clínico Universitario Lozano Blesa (HCULB), San Juan Bosco, 50009 Zaragoza, Spain; 9 Centro de Investigación Biomédica en Red en el Área Temática de Enfermedades Hepáticas y Digestivas (CIBERehd), 28029 Madrid, Spain; 10 Departamento de Ingeniería Electrónica y Comunicaciones, Universidad de Zaragoza, 50009 Zaragoza, Spain

## Abstract

*Background*: Early detection of oncological diseases such as pancreatic ductal adenocarcinoma (PDAC) and ovarian cancer (OV) is pivotal for successful treatment but remains a significant challenge due to the lack of sensitive and specific diagnostic tests. Fluorescence spectroscopy, enhanced by the interaction of serum proteins with nanoparticles (NPs) based on linear–dendritic block copolymers, has emerged as a promising technique for the noninvasive detection of these malignancies. This study introduces a novel array-based assay methodology to evaluate the diagnostic capabilities of various NPs within serum samples using fluorescence. *Methods*: We synthesized three types of NPs (1-SH, 2-OH, 3-NH_3_^+^) and analyzed their fluorescence spectra in serum samples from patients with PDAC, OV, and control subjects. The samples were excited at 330 and 350 nm wavelengths to obtain their fluorescence emission spectra. An array of machine learning algorithms was applied, including boosting and tree-based methods, to assess the ability of the spectral data to discriminate between pathological and nonpathological states. The algorithms’ performance was measured by the area under the receiver operating characteristic curves (AUC). *Results*: The fluorescence spectra revealed distinct patterns for PDAC and OV pathologies. 3-NH_3_^+^ NPs exhibited the highest differential capacity with AUCs exceeding 80% for PDAC across all algorithms, except one. 2-OH NPs showed a strong discriminatory ability for OV with AUCs over 70%, utilizing all but one of the algorithms. 1-SH NPs, however, did not significantly increase differentiability. Boosting algorithms generally outperformed other methods, indicating their suitability for this diagnostic approach. *Conclusions*: The proposed assay array methodology enables the systematic evaluation of NPs’ diagnostic potential using fluorescence spectroscopy. The differential interactions between NPs and serum proteins specific to PDAC and OV highlight the method’s capability to discern pathological states. These findings suggest a path forward for developing NP-assisted fluorescence spectroscopy as a viable tool for cancer diagnostics, potentially leading to earlier detection and improved patient outcomes.

## Graphical abstract

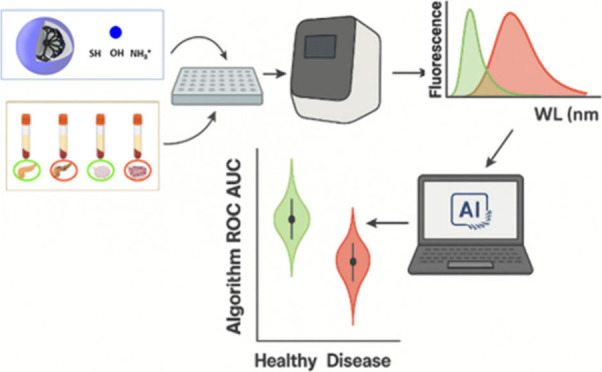

## Introduction

Cancer remains one of the most difficult health challenges of the 21st century, with early detection being pivotal in determining the success of treatment and patient outcomes.^1^ Despite advancements in medical technology, the early diagnosis of many cancers, particularly those with subtle onset and asymptomatic progression like pancreatic ductal adenocarcinoma (PDAC)^2^ and ovarian cancer (OV),^3^ continues to be a significant hurdle. Detecting these malignancies at an early stage can dramatically improve treatment efficacy, reduce mortality rates, and enhance the quality of life for patients.

The current diagnostic landscape for PDAC and OV is fraught with challenges, primarily due to the limitations of existing detection methods. PDAC is notorious for its late diagnosis and high mortality rate, often termed a ’silent killer’ due to its lack of early symptoms. The prognosis for patients with PDAC is particularly dire, with a five-year survival rate that is dismally low, largely due to the advanced stage at which this cancer is typically diagnosed.^4^ Current diagnostic tools for PDAC include imaging techniques such as computed tomography (CT) scans, magnetic resonance imaging (MRI), and endoscopic ultrasound (EUS).^5^ However, these methods often fall short in detecting early stage tumors, as they typically become visible only once they have grown or spread to surrounding tissues. Additionally, the invasive nature of biopsies, often required for a definitive diagnosis, poses risks and discomfort for patients.^4^

Similarly, OV presents a diagnostic challenge. Often labeled as a ’whispering disease’, its symptoms are vague and easily misattributed to less severe health issues. This leads to a majority of OV cases being diagnosed at later stages, when the cancer has already spread, making treatment more complicated and less effective.^6^ Current detection methods for OV, including transvaginal ultrasound and the CA-125 blood test, have limited sensitivity and specificity, particularly for early stage cancer.^7^ The CA-125 test, for example, can yield false positives in noncancerous conditions and may not be elevated in all OV patients, especially in the early stages of the disease.^8^ This results in a significant number of OV cases being diagnosed at an advanced stage, complicating treatment and reducing survival chances.^8^

In this context, the development of innovative diagnostic methodologies that can detect these cancers at their nascent stages is not just desirable but imperative. Our study aims to address this need by exploring the potential of fluorescence spectroscopy, enhanced using nanoparticles (NPs) based on linear dendritic block copolymers (LDBCs), as a novel approach for the early detection of PDAC and OV in serum samples. The following sections will delve into the methodology of this approach and its implications in the realm of cancer diagnostics.

Fluorescence spectroscopy emerges as a promising diagnostic tool, offering several advantages over traditional methods.^9–14^ It is noninvasive, eliminating the risks and discomfort associated with biopsies and other invasive procedures. Furthermore, its integration with nanoparticle technology represents a novel and potentially groundbreaking approach.^15^ NPs can modify the natural fluorescence of biological molecules, increasing the sensitivity and specificity of this method. This advancement holds the promise of detecting subtle biochemical changes in serum samples indicative of early stage malignancies, setting the stage for a significant leap forward in the early detection of cancers like PDAC and OV.

Nanoparticles based on amphiphilic LDBCs offer unique advantages due to their high design flexibility, allowing precision in nanoparticle formation through self-assembly methodologies.^16–19^ In particular, dendritic structures, which are highly branched macromolecules, can be precisely engineered for size and surface functionality.^20^ This customization enables the optimization of their interaction with specific biomolecules found in serum. When introduced into serum samples, these NPs can bind to proteins and other biomolecules altered in the presence of cancer. This binding alters the fluorescence properties of the complex, providing a distinct spectral signature that can be detected and analyzed.^15^

Despite promising advancements in NP-enhanced fluorescence spectroscopy, a significant gap remains in the current research landscape: the lack of a systematic and standardized method to evaluate and compare the diagnostic potential of different NPs. Different NPs can elicit distinct fluorescence responses based on their size, shape, composition, and surface chemistry. These responses are influenced by the biochemical environment within the serum, which varies from one pathological condition to another.^21,22^ Without a standardized method to assess these interactions and their diagnostic implications, the potential of NPs in cancer detection remains only partially tapped.

Addressing this research gap, the main objective of our study is to develop a comprehensive assay array methodology designed to systematically evaluate the fluorescence spectroscopy responses of a range of NPs based on LDBCs (Figure 1) in serum samples under both normal and pathological conditions. By doing so, we aim to establish a versatile and scalable platform that can discern the diagnostic efficacy of various NPs and shed light on their interaction mechanisms with biomolecules in cancerous states.

**Figure 1:**
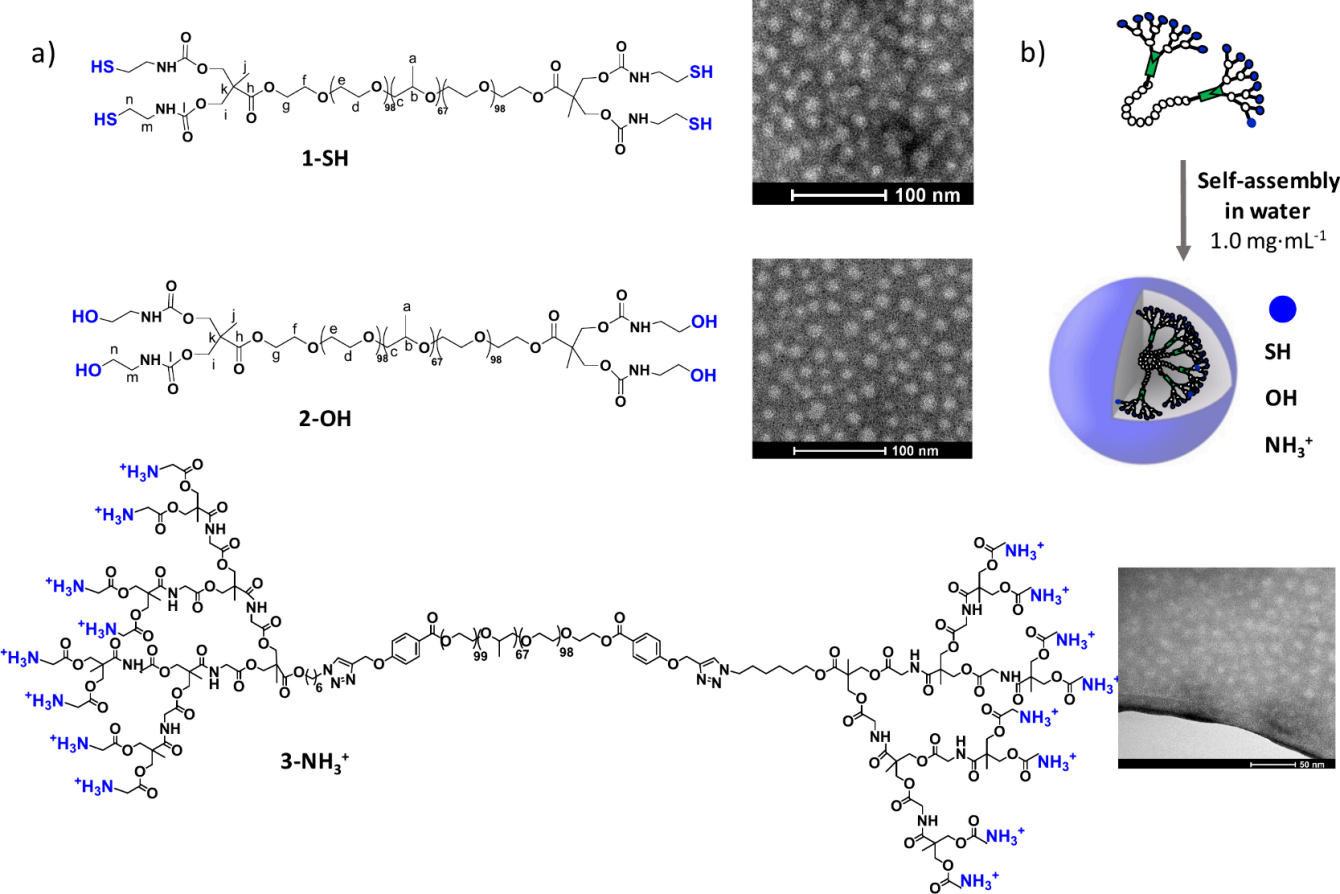
a) Chemical structure of the dendritic polymers used to prepare nanoparticles as shown in transmission electron microscopy (TEM) images. b) Cartoon representation of the formation of nanoparticles with different chemical functionality on the surface by self-assembly in water.

This array-based approach promises several advantages. It enables high-throughput screening of multiple NPs, facilitates direct comparisons of their diagnostic performance, and provides a more nuanced understanding of their interactions with cancer biomarkers. Ultimately, the development of this assay array methodology aims to pave the way for more precise, noninvasive, and effective diagnostic tools in the battle against cancers such as PDAC and OV, fulfilling a critical need in oncological diagnostics.

In conclusion, this study aims to contribute meaningfully to the field of cancer diagnostics by integrating novel methodological approaches with practical, data-driven insights. The potential impact extends far beyond the immediate findings, holding the promise of transforming the landscape of disease detection and management through the innovative use of nanotechnology and fluorescence spectroscopy.

## Materials and Methods

### Synthesis and Characterization of the LDBCs

The synthetic procedures and characterization data of the intermediates are detailed in the Supporting Information. The synthesis of compound 3-NH_3_^+^ was described in a previous work.^23^

## Results

### Synthesis and Morphological Characterization of LDBCs

The linear dendritic block copolymers (LDBCs) employed in this study consist of the conjugation of Pluronic F127, a food and drug administration (FDA) approved poloxamer,^24^ with bis­(hydroxymethyl)­propionic acid (bis-MPA) dendrons,^25^ both widely explored for biomedical applications. Details of the synthesis and analytical data are given in the Experimental Section and in the Supporting Information (Scheme S1). Both compounds 1-SH and 2-OH were synthesized by esterification of both hydroxyl terminal groups of commercial Pluronic F127 with bis­(hydroxymethyl)­propionic acid (bis-MPA) and incorporation of either cysteamine or ethanolamine, respectively, through a carbamate linkage. The synthetic pathway consists of four steps that provide each intermediate with high yields after easy purification processes when needed. The terminal hydroxyl groups of Pluronic F127 were esterified using the benzyl-protected anhydride derived from 2,2-bis­(hydroxymethyl) propionic acid in the presence of DMAP.^26^ Upon cleavage of the benzyl groups by catalytic hydrogenation with Pd/C, the terminal hydroxyl groups of both bis-MPA blocks were made to react with p-nitrophenyl chloroformate using pyridine as a base to give a carbonate derivative, as precursor of the carbamate group. p-Nitrophenol is easily replaced by ethanolamine or cysteamine providing the final carbamate derivatives.

For the formation of NPs by self-assembly, the appropriate volume of distilled water was added to each compound to obtain a final concentration of 1 mg·mL^–1^ in water. The samples were placed 15 min at 4 °C to allow the total dissolution of the derivatives in water, and then they were slowly heated at room temperature. TEM observations showed spherical and monodisperse micelles, with average diameters calculated around 17 ± 2 nm for 1-SH and 13.5 ± 2 nm for 2-OH (Figure 1b).

3-NH_3_^+^ is a cationic LDBC, which was reported by our group to form micelles of nanometric size that proved to be efficient as drug-carriers for antimalarial drugs. This polymer, which was synthesized by copper-catalyzed azide–alkyne cycloaddition (click chemistry), forms spherical micelles with a periphery of ammonium groups, and a size of 13 ± 3 nm, as determined by TEM.^23^ Its behavior so as to interact with proteins in the blood serum was expected to be similar to that previously published by us.^15^

### Fluorescence Spectra of PDAC and OV from Patients’ Serum Samples

Three distinct NPs (1-SH, 2-OH and 3-NH_3_^+^) were tested and serum samples from two different pathologies: pancreatic ductal adenocarcinoma (PDAC) and ovarian cancer (OV) and their respective controls were studied. Samples were placed in a microplate in the way it is described in Figure 2.

**Figure 2:**
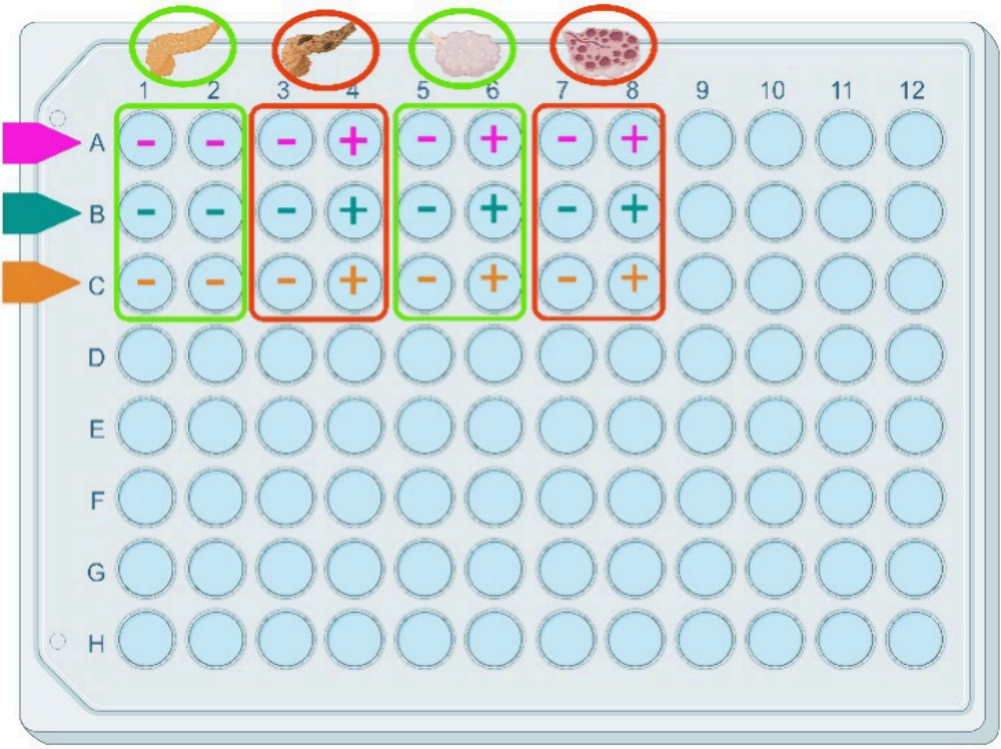
Assay plate layout for the evaluation of three different nanoparticles (NPs) in the detection of pathological conditions. Rows A, B, and C represent the three NP types: **1-SH** (Pink), **2-OH** (dark green), and **3-NH3** ^**+**^ (orange), respectively. The (±) signs denote the presence or absence of NPs in the sample. Columns 1 and 2 correspond to blood donors control group (BD), columns 3 and 4 correspond to Pancreatic Ductal Adenocarcinoma (PDAC), columns 5 and 6 correspond to benign cyst control group (OC) and columns 7 and 8 correspond to Ovarian Cancer (OV). Created in BioRender. Abian, O. (2025) https://BioRender.com/pvv6wnb.

By utilizing the raw fluorescence curves, it becomes feasible to calculate the differential fluorescence curves that compare the samples with and without the presence of NPs. These differential curves provide crucial insights into the influence of NPs on the fluorescence spectra, enabling a comprehensive analysis of the sample characteristics (Figure 3).

**Figure 3:**
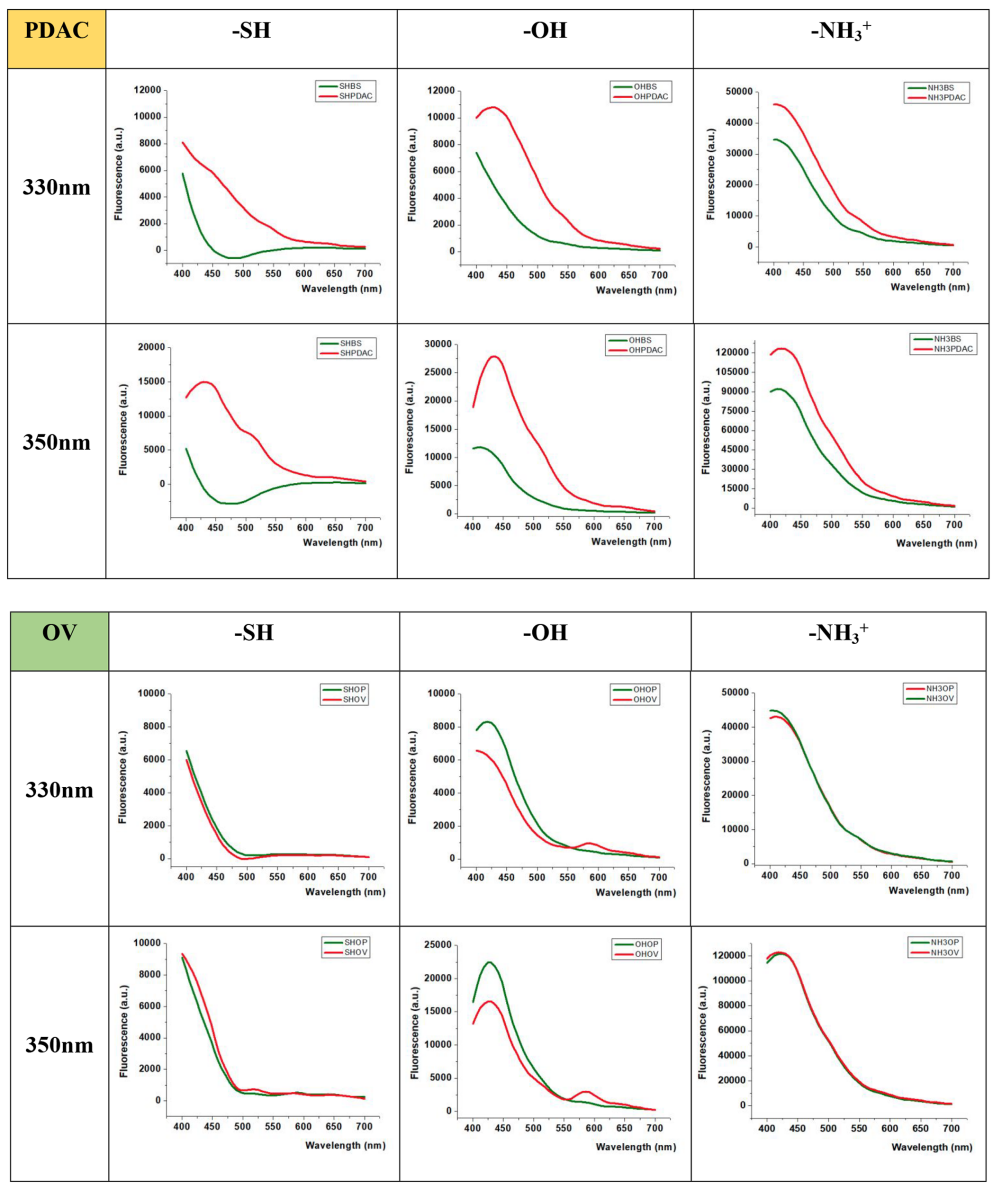
Mean of differential Fluorescence Spectra curves (obtained by subtracting the signal without NP to the signal with NP) of serum samples pancreatic ductal adenocarcinoma (PDAC) cancer and ovarian cancer (OV) (red lines), and their corresponding controls or nondiseased individuals in each case (green lines). NP sample Serum was diluted 1:25 in phosphate buffered saline, with NP concentration of 500 μg·mL^–1^, the excitation wavelength was 330 or 350 nm, and the emission spectra was registered from 400 to 700 nm.

### Machine Learning Algorithms for Discriminating Healthy and Pathological Samples Based on Differential Fluorescence Signal Data with and without NPs

In an extensive comparative study, the performance of various machine learning algorithms was assessed for the detection of PDAC and OV using fluorescence spectra excited at 330 and 350 nm: AdaBoost, CatBoost, DecissionTree, GaussianNB, KNeighborsClassifier, Logistic Regression, Random Forest, SVM and XGBoost. The results are summarized in Table 1 and Table 2 for AUC metric.

**Table 1:** ROC-AUC Values for the Different Algorithms Applied to PDAC Samples Excited at 330 and 350 nm and Their Corresponding Standard Deviation for the Different Instances of the Model in K-Fold Implementation^a^

	330 nm						350 nm					
	1-SH		2-OH		3-NH_3_^+^		1-SH		2-OH		3-NH_3_^+^	
PDAC study	global ROC-AUC	Std deviation	global ROC-AUC	Std deviation	global ROC-AUC	Std deviation	global ROC-AUC	Std deviation	global ROC-AUC	Std deviation	global ROC-AUC	Std deviation
AdaBoost	0.690	0.110	0.771	0.113	***0.920***	0.057	0.771	0.129	0.717	0.110	0.649	0.171
CatBoost	0.685	0.135	0.672	0.074	0.920	0.066	0.713	0.108	0.692	0.103	0.619	0.122
XGBoost	0.744	0.115	0.783	0.131	0.918	0.060	***0.791***	0.119	0.723	0.089	0.670	0.134
DecissionTree	0.640	0.081	0.695	0.104	0.825	0.086	0.665	0.132	0.630	0.077	0.596	0.135
Random Forest	0.717	0.145	0.770	0.117	0.901	0.084	0.740	0.116	0.711	0.090	0.680	0.139
GaussianNB	0.716	0.119	0.720	0.103	0.872	0.088	0.763	0.098	0.710	0.136	0.632	0.132
KNeighborsClassifier	0.698	0.138	0.775	0.100	0.888	0.060	0.763	0.107	0.671	0.101	0.592	0.122
SVM	0.520	0.205	0.690	0.119	0.883	0.074	0.575	0.138	0.618	0.091	0.369	0.122
Logistic Regression	0.689	0.140	0.700	0.106	0.737	0.101	0.781	0.081	0.663	0.067	0.616	0.108

a The italic text indicates the cell with the highest value of the global ROC-AUC value for that excitation wavelength, and the bold text indicates the highest value for the specific column.

**Table 2:** ROC-AUC Values for the Different Algorithms Applied to OV Samples Excited at 330 and 350 nm and Their Corresponding Standard Deviation for the Different Instances of the Model in K-Fold Implementation^a^

	330 nm						350 nm					
	1-SH		2-OH		3-NH_3_^+^		1-SH		2-OH		3-NH_3_^+^	
OV study	global ROC-AUC	Std deviation	global ROC-AUC	Std deviation	global ROC-AUC	Std deviation	global ROC-AUC	Std deviation	global ROC-AUC	Std deviation	global ROC-AUC	Std deviation
AdaBoost	0.504	0.084	0.680	0.069	0.763	0.100	0.619	0.143	0.891	0.085	0.573	0.136
CatBoost	0.462	0.107	0.752	0.147	0.716	0.138	0.553	0.158	0.877	0.099	0.525	0.143
XGBoost	0.539	0.123	0.716	0.101	***0.769***	0.137	0.587	0.145	***0.901***	0.080	0.629	0.187
DecissionTree	0.531	0.097	0.656	0.067	0.582	0.150	0.575	0.110	0.817	0.119	0.551	0.140
Random Forest	0.520	0.125	0.737	0.128	0.679	0.192	0.570	0.089	0.870	0.058	0.662	0.141
GaussianNB	0.524	0.115	0.764	0.111	0.505	0.155	0.506	0.103	0.831	0.062	0.610	0.154
KNeighborsClassifier	0.551	0.131	0.737	0.109	0.652	0.148	0.490	0.126	0.880	0.091	0.591	0.140
SVM	0.349	0.132	0.401	0.275	0.498	0.204	0.457	0.159	0.417	0.192	0.347	0.186
Logistic Regression	0.484	0.107	0.642	0.175	0.701	0.148	0.650	0.130	0.673	0.129	0.601	0.106

a The italic text indicates the cell with highest value for that excitation wavelength, and the bold text indicates the highest value for the specific column.

The remaining metrics analyzed exhibit values comparable to the area under the ROC-AUC, as can be seen in Table S1 and Table S2. Since the objective is not to focus on any specific aspect but rather to achieve a general evaluation of the classification performance of the models, it has been decided to primarily work with the AUC parameter. Nevertheless, the other metrics remain available for reference or for further in-depth analysis if required.

Figure 4 clusters the ROC-AUC values of the algorithms according to different NPs for PDAC pathology. The values of ROC-AUC present good diagnosis performance in general, especially for 3-NH_3_^+^ at 330 nm. In contrast, at 350 nm, this NP presents poorer performance than the other NPs, whereas these have similar values (better for 1-SH than for 2-OH).

**Figure 4:**
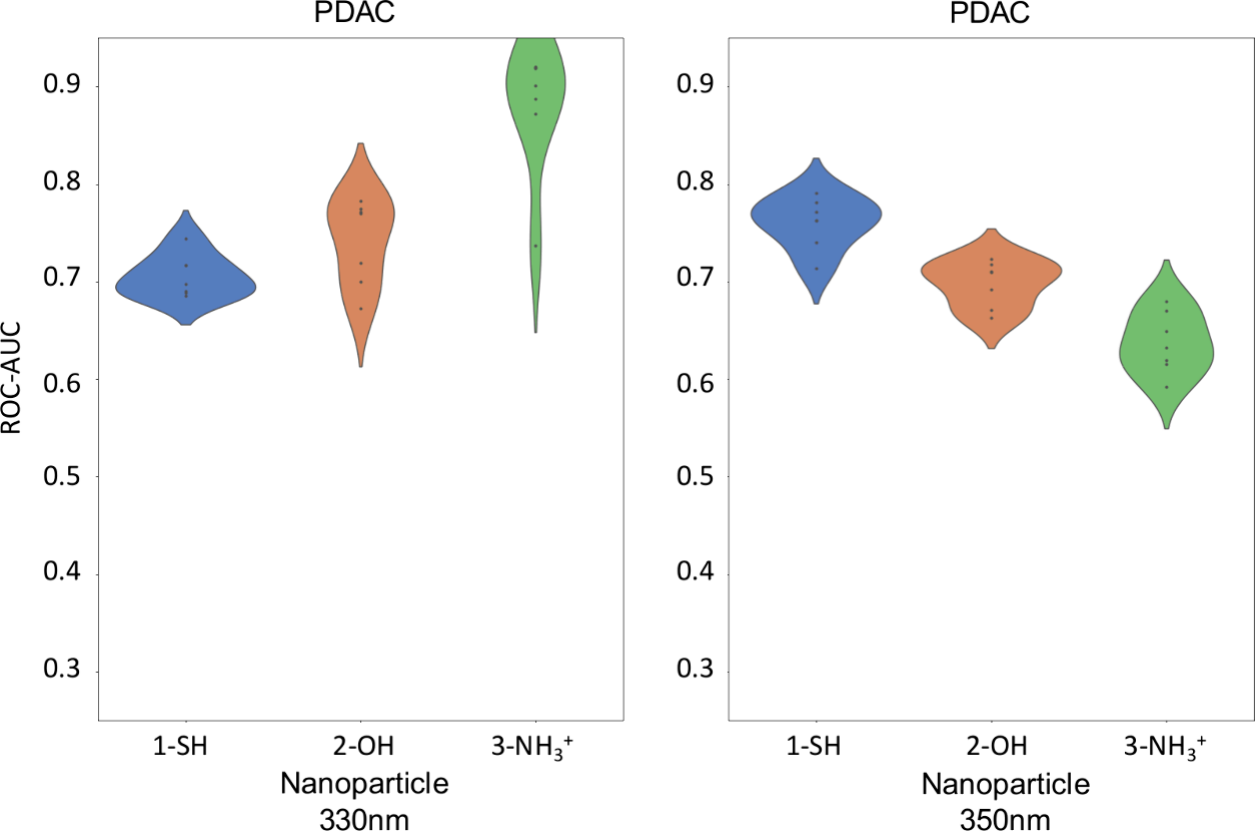
Violin plots of the area under the receiver operating characteristic curve values for pancreas cancer study the different algorithms, excited at 330 nm (left) and 350 nm (right).

Figure 5 collects the ROC-AUC values for OV pathology, where 2-OH shows very good diagnosis performance, especially at 350 nm. 1-SH presents poor performance in general, except for some specific algorithm. 3-NH_3_^+^ displays very different performance depending on the wavelength and algorithm combination. Particularly noteworthy in this case is the performance of some algorithms and the 350 nm wavelength combination with 2-OH.

**Figure 5:**
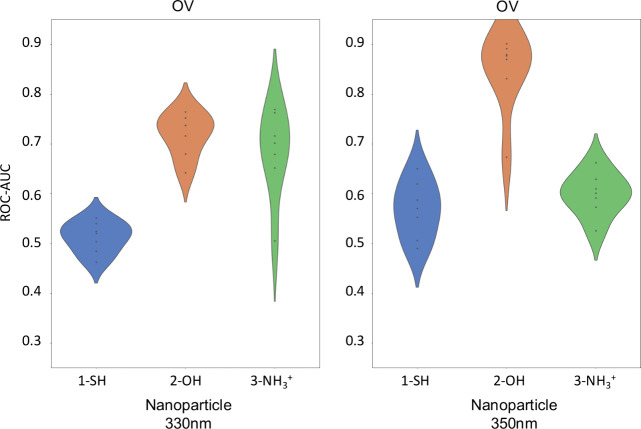
Violin plots of the area under the receiver operating characteristic curve values for ovarian cancer study the different algorithms, excited at 330 nm (left) and 350 nm (right).

Table 1 and Table 2 show that standard deviations are in general around 0.1, being very much higher for lower ROC-AUC values, reaching 0.205 (for SVM algorithm for PDAC with 1-SH at 330 nm) and 0.275 (for SVM algorithm for OV with 2-OH at 330 nm), getting in both cases a poor diagnosis performance. On the other hand, 3-NH_3_^+^ induces greater variability in the results of the algorithms for both pathologies, with some achieving superior performance, while others yield results inferior to those obtained with other NPs. 2-OH induces good diagnosis performance in almost all cases, and 1-SH gets better results in PDAC pathology.

In the evaluation of NP-aided fluorescence spectroscopy for the detection of pathological conditions, our results elucidate the intricate interplay between the type of NP, the machine learning algorithm used, and the pathological condition under investigation. Rather than presenting a universal trend, the effectiveness of each NP-algorithm pairing emerged as highly specific to the condition being diagnosed.

The results can be categorized by NP type to elucidate the comparative performance in detecting PDAC and OV.

#### 1-SH Nanoparticles

For PDAC study, this NP does not yield poor results across all the models used, although there is room for improvement. The highest AUC value is 0.791, obtained with the XGBoost at a wavelength of 350 nm, while many other values are around 0.74. At 330 nm, the results are generally worse, but still around 0.70. In the context of OV study, results are unsatisfactory across all models (only two models reach an AUC of 0.60), indicating that this NP does not contribute to the differentiation needed for the detection of this disease.

#### 2-OH Nanoparticles

2-OH NPs showed a strong affinity for PDAC detection when paired with the XGBoost algorithm at 330 nm, achieving an ROC-AUC of 0.783, that could not be improved when exciting samples at 350 nm (reaching 0.723). For OV study, 2-OH NPs again excelled with the XGBoost algorithm at 350 nm excitation, where they achieved a ROC-AUC of 0.901. Lower results were obtained exciting at 330 nm with GaussianNB Algorithm (0.764) as the best combination. In general, these 2-OH NPs in OV exhibited very good performances at both excitation wavelengths and algorithms, especially for 350 nm. This illustrates the versatility of 2-OH NPs for both pathologies across different excitation wavelengths and their robustness with all algorithms, but specially with ensemble learning algorithms highlighting their potential as a diagnostic tool for OV.

#### 3-NH_3_^+^ Nanoparticles

3-NH_3_^+^ NPs, when utilized with the AdaBoost algorithm at 330 nm, showed a notable ROC-AUC of 0.920 for PDAC. At 350 nm, the results are generally worse, not reaching 0.7 with any algorithm. These NPs exhibited the best performance in PDAC at 330 nm wavelength for all the algorithms tested, except for LogisticRegression. On the other hand, for 330 nm, the results are worse than for other NPs. This suggests a strong predictive capability that could be harnessed in a clinical setting for early PDAC detection.

For OV study, 3-NH_3_^+^ NPs did not reach the same level of efficacy as of PDAC with any of the algorithms tested at 330 and 350 nm. The best algorithm at 330 nm with a ROC-AUC of 0.769 was XGBoost (which was similar to the worst result in case of PDAC, confirming again that it is important to attend both NP and algorithm, when setting up the best diagnosis protocol).

The results can also be analyzed attending the different algorithms to compare their performances. In general, Boost ensemble algorithms have demonstrated strong discriminative capabilities, consistently achieving some of the best results.

The data underscores the importance of tailored NP-algorithm pairings, with certain combinations emerging as particularly effective for specific pathologies. These findings pave the way for precision diagnostics, where the choice of the NP and the machine learning strategy is critical to achieving high diagnostic accuracy.

Figure 6 and S2 display a comprehensive suite of ROC curves and probability distribution histograms, offering a visual comparison of the diagnostic performance of various machine learning algorithms when paired with different NPs in identifying PDAC and OV. These analyses are segmented based on the excitation wavelengths employed during fluorescence spectroscopy, specifically at 330 and 350 nm, revealing the nuanced effects of excitation wavelength on the efficacy of cancer detection.

**Figure 6:**
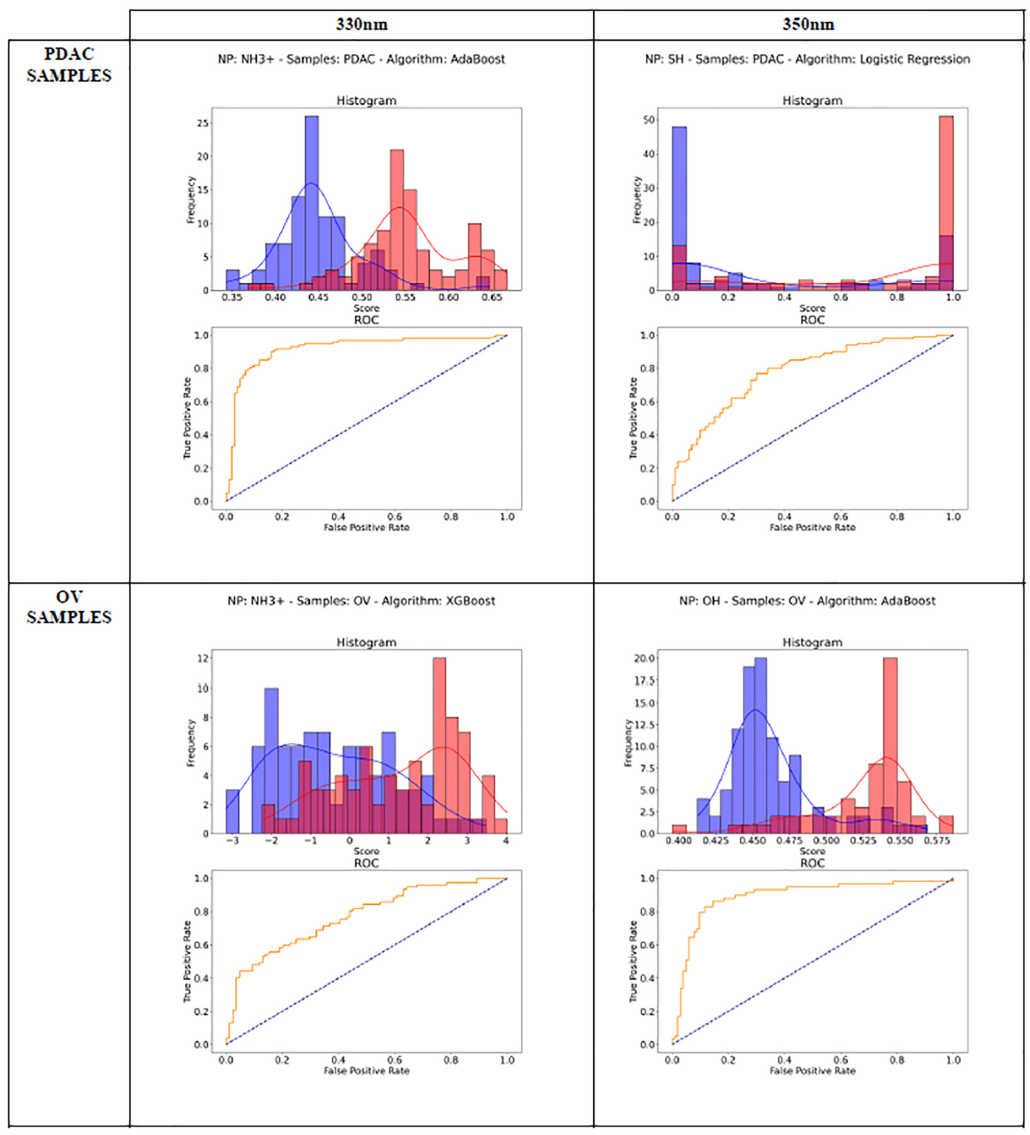
Comparative ROC Curve Analysis and Probability Distribution Histograms for NP-Assisted Fluorescence Spectroscopy in Pathology Detection. Left column results for 330 nm excitation wavelength, and the right one for 350 nm. Each subplot corresponds to the best combinations of a specific machine learning algorithm and nanoparticle (NP) type: (from top to bottom and from left to right) AdaBoost with NH3^+^, Logistic Regression with SH; XGBoost with NH3^+^, AdaBoost with OH. ROC curves are plotted in the insets, with true positive rates (sensitivity) on the *y*-axis against false positive rates (1-specificity) on the *x*-axis. The AUC values are indicative of the model’s ability to discriminate between the pathological and nonpathological states. The histograms exhibit the distribution of predicted probabilities for pathological (red bars) and nonpathological (blue bars) conditions, with a clear separation suggesting a higher model performance.

Each ROC curve within the Figure 6, serves as an indicator of the true positive rate versus the false positive rate for the respective algorithm-NP combination, with the ideal model showing a curve that pushes toward the upper left corner, signifying a higher AUC and, consequently, better diagnostic performance. The diagonal line represents the baseline performance of a random classifier. The histograms complement the ROC curves by detailing the distribution of the predicted probabilities for the pathological (positive) and nonpathological (negative) classes, with the blue bars typically denoting the negatives and the red bars the positives. A more pronounced separation between these distributions correlates with a more accurate model.

#### Algorithm and NP Efficacy

The efficacy of different algorithms in conjunction with specific NPs can be discussed by highlighting which combinations show the greatest separation between the positive and negative class distributions, as evidenced by the histogram plots, and the largest AUC values. For instance, at 330 nm, the 3-NH_3_^+^ nanoparticles when analyzed with AdaBoost showcases a significant discriminative capability for PDAC, as reflected by its ROC curve and well-separated probability histograms (Figure 6).

#### Differences among Pathologies

The Figure 6 and S1 also allows for an examination of how NP-algorithm combinations vary in their diagnostic capabilities across different pathologies. One might note, for instance, that 3-NH_3_^+^ nanoparticles paired with the AdaBoost algorithm exhibit a strong AUC for PDAC at 330 nm, whereas the combination’s performance may differ when applied to OV detection.

## Discussion

This present study explores the diagnostic potential of nanoparticles (NPs) based on linear dendritic block copolymers (LDBCs) through fluorescence spectroscopy, which has yielded significant insights. By integrating nanotechnology with machine learning, we have developed a promising approach for the early detection of pancreatic ductal adenocarcinoma (PDAC) and ovarian cancer (OV).

The differential behavior of a single NP type across various pathologies underscores the complex nature of disease-specific fluorescence signatures. Pancreatic samples, PDAC, exhibited distinguishable characteristics with AUC ROC values exceeding 60% across almost all algorithm-NP pairings. Notably, 3-NH_3_^+^ nanoparticles demonstrated heightened detectability for pancreatic pathology, achieving ROC-AUC values greater than 80% across nearly all algorithms. This pronounced interaction suggests that 3-NH_3_^+^ NPs are particularly effective in highlighting the biochemical changes associated with PDAC. Conversely, ovarian samples showed comparable levels of differentiation with 2–OH nanoparticles, achieving ROC-AUC values above 70% with most algorithms, indicating their potential utility in OV diagnostics.

The clinical relevance of these findings can be interpreted as high AUC values for PDAC detection suggesting a potential for early disease identification, and this is critical due to the typically late diagnosis and poor prognosis associated with pancreatic cancer. Similarly, the algorithm-NP pairings that demonstrate superior performance in OV detection could be indicative of a method that may enhance screening and diagnostic processes for OV. A restriction of the present study is the sex imbalance between PDAC and control cohorts, which may introduce confounding effects. However, stratified analysis indicated that the spectral differences observed are independent of sex (see SI). Future studies should consider sex-matched cohort designs to minimize potential bias.

From the perspective of nanoparticles, 3-NH_3_^+^ NPs revealed a distinct differentiating effect, especially in pancreatic samples, achieving notable discrimination. The 2–OH NPs consistently demonstrated detectable differentiation between malignant and nonmalignant samples in both ovarian and pancreatic cases, with their effect being particularly remarkable in ovarian samples, where only a few algorithms paired with 3-NH_3_^+^ achieved similar values. The 1–SH nanoparticles, however, did not enhance differentiability to a notable extent, suggesting a need for further investigation into their interaction dynamics with serum proteins.

The discriminative power of the algorithms varied for the same NP and pathology. ’Boosting’ combination algorithms, such as AdaBoost, CatBoost, and XGBoost, generally yielded superior results. Lastly, the results can inform further refinement of machine learning models. Based on the probability histograms, adjustments to classification thresholds could be considered to improve the sensitivity or specificity of the diagnostic models. This could involve increasing the threshold for classifying a sample as pathological if the aim is to reduce false positives or lowering it to capture more true positives at the expense of increasing false positives, depending on the clinical priorities.

Achieving the best possible outcome in certain identifiable groups proved more crucial than performing consistently across all NP-disease combinations. For example, in the 3-NH_3_^+^ pancreatic sample group at 330 nm, some algorithms were capable of obtaining ROC-AUC values greater than 0.90, with XGBoost, AdaBoost, Random Forest, and CatBoost showing particularly high effectiveness.

General observations highlight that the presence of NPs with diverse structures within serum samples perturbs the fluorescence spectrum of serum proteins in unique ways, reflecting the varied interactions between the NPs and the proteins. This ability to generate differential fluorescence spectra in serum samples paves the way for distinguishing between pathological and nonpathological states.^15^ The observed fluorescence changes are likely driven by complex NP–protein interactions, including the formation of a protein corona that differs between healthy and cancerous serum. These changes may involve not only direct binding events but also protein conformational rearrangements or altered quenching environments around the NP surface. Although the NPs used are not designed to target specific proteins, the resulting spectral fingerprints reflect cumulative disease-related alterations in serum proteome composition and structure. Rather than targeting individual biomarkers, our array-based system exploits this emergent pattern as a diagnostic signature. This strategy, based on differential pattern recognition, provides robustness in the presence of complex biological background and minimizes the need for extreme specificity at the molecular level. However, we acknowledge that a full proteomic characterization of the protein corona and identification of specific biomarkers responsible for the signal remains a critical next step. Future work will integrate mass spectrometry-based proteomics to validate the protein-level interactions and explore their diagnostic significance.

The analysis of these differential fluorescence spectra using machine learning algorithms has the potential to evolve into a viable diagnostic tool. By harnessing the power of machine learning to interpret these spectral changes, we can edge closer to realizing precision diagnostics that differentiate between health and disease states with unprecedented accuracy.

One of the key contributions of this study is the establishment of a versatile framework that can systematically evaluate the diagnostic potential of various nanoparticles. This framework has the capability to differentiate between pathological and nonpathological serum samples with a high degree of accuracy, particularly for challenging cancers such as PDAC and OV. The ability to detect these cancers at an early stage through a minimally invasive serum test could dramatically shift the current diagnostic paradigm, leading to earlier interventions and potentially improved patient outcomes.

Moreover, the methodology developed in this study is not limited to PDAC and OV. Its principles and techniques can be readily adapted to other types of cancer and diseases. The flexibility of the array design allows for the testing of NPs with different peripheral chemical groups and the exploration of their interactions with a wide range of biomarkers. Similarly, the machine learning algorithms can be trained and validated on diverse data sets, making them applicable to various pathological conditions.

The integration of nanotechnology and machine learning in fluorescence spectroscopy could also spur innovation in other diagnostic areas. For instance, this approach might be valuable in monitoring treatment response or in detecting recurrence, offering a rapid and noninvasive alternative to traditional methods. Furthermore, the insights gained into the interactions between NPs and serum components could inform the development of targeted therapies and personalized medicine approaches.

One limitation of the current study is the absence of direct biological imaging to support the observed fluorescence spectral changes. As our method is based on label-free serum analysis, it does not involve tissue or cellular visualization. However, future studies are planned to integrate complementary imaging techniques such as immunohistochemistry or fluorescence microscopy to further validate nanoparticle–biomolecule interactions at the tissue level.

The quest for early and accurate detection of cancerous pathologies has been a driving force behind numerous advancements in biomedical diagnostics. Early detection of malignancies such as PDAC and OV remains a difficult challenge in clinical oncology. The subtle onset and often asymptomatic nature of these diseases underscore the need for innovative diagnostic techniques that offer both sensitivity and specificity. In recent years, the advent of NP-enhanced fluorescence spectroscopy has emerged as a promising approach to address this challenge. This technique leverages the unique optical properties of NPs to enhance the fluorescence signals from biological samples, potentially allowing for the early detection of cancerous changes in serum.

Compared to recent studies that apply machine learning to cancer diagnostics,^27–29^ our approach offers several distinctive advantages. While many of these studies rely on the quantification of individual molecular biomarkers or omics data, our method is based on global pattern recognition derived from the fluorescence response of serum exposed to a nanoparticle array. This label-free and nontargeted strategy reduces the need for preidentified biomarkers and allows for a more flexible and accessible diagnostic framework. In addition, our implementation of ensemble machine learning models such as XGBoost and CatBoost yielded superior classification performance, particularly in challenging pathologies such as PDAC.

The interplay between NPs, machine learning algorithms, and disease-specific fluorescence patterns offers fertile ground for further research and development of diagnostic methodologies. As we continue to refine these techniques and deepen our understanding of their underlying principles, the prospect of enhancing early detection and diagnosis of malignancies through fluorescence spectroscopy becomes increasingly tangible. The tailored NP-algorithm combinations highlighted in this research represent a significant step forward in the quest for more effective, noninvasive cancer diagnostic tools.

These results underscore the importance of selecting the right combination of algorithm and NP, as well as the optimal excitation wavelength, to achieve the most accurate and clinically useful diagnostic results.

## Conclusions

This study has demonstrated the significant potential of integrating nanoparticles (NPs) based on linear–dendritic block copolymers with fluorescence spectroscopy and machine learning algorithms for the early detection of pancreatic ductal adenocarcinoma (PDAC) and ovarian cancer (OV). The results indicate that this approach could revolutionize noninvasive cancer diagnostics.

The synthesized nanoparticles—1–SH, 2–OH and 3-NH_3_^+^—show remarkable capabilities in distinguishing between malignant and nonmalignant serum samples. 3-NH_3_^+^ NPs, in particular, showed high effectiveness in detecting PDAC, while 2–OH NPs excelled in identifying OV. This highlights the importance of selecting appropriate NPs tailored to specific cancer types to achieve optimal diagnostic performance.

The study revealed that ’boosting’ algorithms such as AdaBoost, CatBoost, and XGBoost generally provided superior diagnostic accuracy. The right NP-algorithm combination is crucial, as different pairings yielded varying levels of efficacy depending on the cancer type and excitation wavelength used.

A key contribution of this research is the establishment of a versatile diagnostic framework. This method can systematically evaluate the diagnostic potential of various nanoparticles, reliably differentiating pathological from nonpathological serum samples with high accuracy. This framework is particularly significant for challenging cancers like PDAC and OV, where early detection is crucial.

In conclusion, the integration of nanotechnology and machine learning in fluorescence spectroscopy offers a promising avenue for developing sensitive, specific, and noninvasive diagnostic tools. These advancements have the potential to significantly improve early detection rates and patient outcomes, paving the way for future innovations in precision medicine.

## Supplementary Material



## Notes

Synthesis and chemical characterization of 1-SH and 2-OH; Characterization techniques; Subject and samples; Blood sample processing; Fluorescence spectroscopy; Machine Learning algorithms; Comparative ROC Curve Analysis and Probability Distribution Histograms for NP-Assisted Fluorescence Spectroscopy in Pathology Detection; Tables for the different evaluation values for the different algorithms (PDF)

## ABBREVIATIONS

AUC: area under the curve
*bis*-MPA: 2,2′-*bis*(hydroxymethyl)­propionic acid
CA-125: cancer antigen 125
CT: computed tomography
DCM: dichloromethane
DMAP: 4-(dimethylamino)­pyridine
EUS: endoscopic ultrasound
HS: human serum
HSA: human serum albumin
LDBC: linear dendritic block copolymer
MALDI-TOF: matrix assisted laser desorption/ionization – Time of flight
MRI: magnetic resonance imaging
NMR: nuclear magnetic resonance
NPs: nanoparticles
OC: ovary cysts
OV: ovary cancer
PBS: phosphate buffer saline
PDAC: pancreatic ductal adenocarcinoma
ROC curve: receiver operating characteristic curve
SVM: support vector machine
TEM: transmission electron microscopy
