# Supplementary material for: Serum-Based Detection of Pancreatic and Ovarian Cancer via a Nanoparticle-Enhanced Fluorescence Array and Machine Learning
Source: Anal Chem. 2025 Jun 23;97(26):13850–60. doi: 10.1021/acs.analchem.5c00974 (PMC12242897; doi:10.1021/acs.analchem.5c00974)
Supplement: Supplementary file 1 [file ac5c00974_si_001.pdf]

Serum-Based Detection of Pancreatic and Ovarian Cancer via a Nanoparticle-Enhanced Fluorescence Array and Machine Learning

Violeta Morcuende-Ventura[1,2†], Oscar Sánchez-Gracia[3†], Natalia Abian-Franco[4], Isabel Jiménez-Pardo[1,2], Lucía Herrer[1,2], Martín Castillo-Vallés[1,2], Alexandre Lancelot[1,2], F. Javier Falcó-Martí[5,6], Sonia Hermoso-Durán[5,6], Roberto Pazo-Cid[6,7], Ángel Lanas[3,6,8,9], Adrián Velazquez-Campoy[3,5,6,9], Teresa Sierra[1,2*] and Olga Abian[3,5,6,9*].

[1]Instituto de Nanociencia y Materiales de Aragón (INMA), CSIC-Universidad de Zaragoza, 50009 Zaragoza, Spain.

[2]Departamento de Química Orgánica, Facultad de Ciencias, Universidad de Zaragoza, 50009 Zaragoza, Spain.

[3]Departamento de Bioquímica y Biología Molecular y Celular, Universidad de Zaragoza, 50009 Zaragoza, Spain.

[4]Hospital Reina Sofía, Carr. Tarazona, Km. 4, 31500, Tudela, Navarra, Spain.

[5]Institute of Biocomputation and Physics of Complex Systems (BIFI), Universidad de Zaragoza, Zaragoza, 50018, Spain.

[6]Instituto de Investigación Sanitaria Aragón (IIS Aragón), 50009, Zaragoza, Spain.

[7]Hospital Universitario Miguel Servet (HUMS); Paseo Isabel la Católica, 1-3, 50009 Zaragoza.

[8]Hospital Clínico Universitario Lozano Blesa (HCULB), San Juan Bosco, 50009 Zaragoza.

[9]Centro de Investigación Biomédica en Red en el Área Temática de Enfermedades Hepáticas y Digestivas (CIBERehd), 28029, Madrid, Spain.

**Synthesis and characterization of 1-SH** and **2-OH.**	**S2**
**Characterization techniques.**	**S4**
**Subjects and samples.**	**S4**
**Blood sample processing.**	**S5**
**Fluorescence spectroscopy.**	**S5**
**Machine Learning Algorithms.**	**S7**

Synthesis and characterization of 1-SH and **2-OH.**

Pluronic F127® (Average Mw= 12600), 2,2-bis(hydroxymethyl)propionic acid (bis-MPA), dimethylaminopyridine (DMAP), Pd/C 10%, p-nitrophenyl chloroformate, cysteamine and ethanolamine were obtained from Sigma-Aldrich. Pluronic F127® was dried during 3 hours at 100 ^{o}C under vacuum prior to use. All solvents were purchased from Sigma-Aldrich.

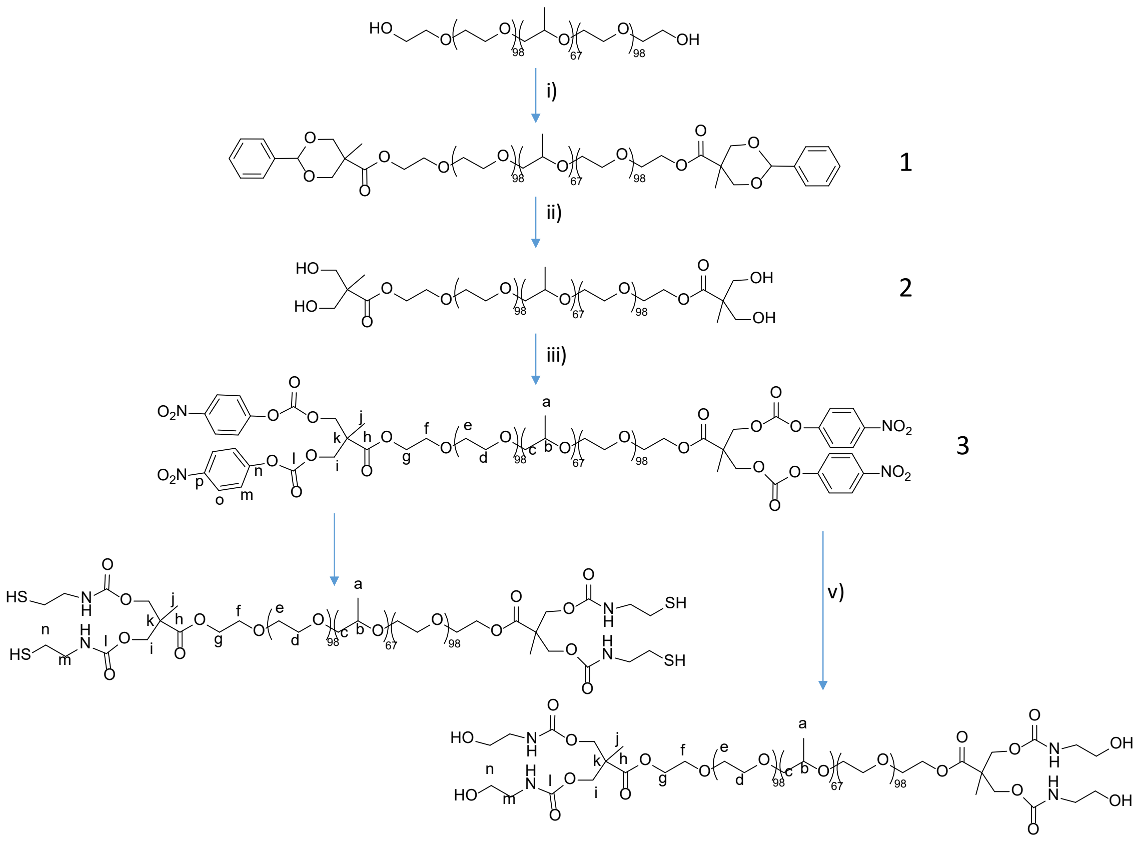

Scheme S1. Synthesis of 1-4[SH] and 2-4[OH]. Reagents and reaction conditions: (i) benzylidene-2,2-bis(oxymethyl)propionic anhydride, DMAP, DCM, r.t., overnight; (ii) Pd/C/H_2, ethyl acetate, r.t. overnight; (iii) p-nitrophenyl chloroformate, pyridine, DCM, argon atmosphere, r.t., overnight; iv) cysteamine, DCM, argon atmosphere, r.t. overnight; v) ethanol amine, DCM, argon atmosphere, r.t. overnight.

Compound 1. Pluronic F127® (10.0 g, 0.79 mmol) was dried at 100 ^{o}C for 2h and dissolved in dry DCM (20 mL). DMAP (0.12 g, 0.95 mmol) and benzylidene-2,2-bis(oxymethyl)propionic anhydride (2.0 g, 4.76 mmol) were added. The mixture was stirred at room temperature overnight. The excess of anhydride was quenched by adding methanol (6 mL). The mixture was stirred overnight and the crude was precipitated in cold diethyl ether (1 L). The product was isolated as a white powder after filtration. Yield: 95 %. **^{1}H-NMR** (400 MHz, $CDCl_3$, δ): 1.04 (s, 6H, -C*H*$_3$), 1.13 (m, 201H, -C*H*$_3$), 3.37-3.82 (m, ~1100H, -O-C*H*$_2$-C*H*$_2$-O-), 4.35 (m, 4H, -CH$_2$-C*H*$_2$-OC(O)-), 4.66 (d, *J*=11.6 Hz, 4H, -C*H*$_2$-OC(O)-), 5.44 (s, 2H, -C*H*-Ph), 7.32 (m, 6H, Ar H), 7.42 (m, 4H, Ar H); **^{13}C-NMR** (100 MHz, $CDCl_3$, δ): 17.3, 17.4, 17.9, 42.4, 64.2, 68.5, 68.6, 69.1, 70.5, 72.9, 73.3, 75.1, 75.3, 75.5, 101.7, 126.2, 128.2, 128.9, 137.9, 173.9; **MALDI^{+}** maximum m/z 5050.2 and 13297.2.

Compound 2. Compound **1** (9.5 g, 0.73 mmol) was dissolved in ethyl acetate (200 mL). Then, Pd/C (10 % by weight) was added. After three vacuum-argon cycles, the reaction mixture was stirred at room temperature in hydrogen atmosphere overnight. The Pd/C was filtered off with Celite® and the filtrate was evaporated to give a white solid. Yield: Quant.

^{1}H NMR (300 MHz, $CDCl_3$, δ): 1.11 (s, 6H, -C*H*$_3$), 1.13 (m, 201H, -C*H*$_3$), 3.37-3.82 (m, ~1100H, O-C*H*$_2$-C*H*$_2$-O-), 4.34 (m, 4H, G, -CH_2-C*H*$_2$-OC(O)-); **^{13}C NMR** (75 MHz, $CDCl_3$, δ): 17.1, 17.3- 17.4, 49.5, 63.2, 67.3, 68.7, 70.5, 72.9, 73.3, 75.1, 75.3, 75.5, 175.6; **MALDI$^+$**: maximum m/z 5213.9 and 13780.1;

Compound 3. Compound **2** (2.3 g, 0.18 mmol) was weighed into a flask and dissolved in 17 mL of dry DCM. Then dry pyridine (0.8 mL) and p-nitrophenyl chloroformate (0.6 g, 2.9 mmol) were added under argon atmosphere. The reaction was kept at room temperature with stirring overnight. The reaction crude was dissolved in 50 mL DCM, and washed with 1 M $NaHSO_4$ (2 x 30 mL) and saturated NaCl solution (1 x 30 mL). The organic phase was dried with $MgSO_4$ and concentrated at the rotary evaporator. The concentrate was poured into 200 mL of cold diethyl ether. The precipitated product was obtained as a white solid after filtration and washing with cold diethyl ether. Yield: 79%.
^{1}H-RMN (300 MHz, CDCl3): δ (ppm): 1.13 (m, 201H, a), 1.38 (s, 6H, J), 3.37-3.85 (m, ~1100H, b, c, d, e, f), 4.34 (m, 4H, g), 4.52 (*AB*q, 8H, ΔvAB=31.8 Hz, *JAB*= 9 Hz, i), 7.37 (m, 8H, m), 8.26 (m, 8H, o). **^{13}C-RMN** (75 MHz, CDCl3): δ (ppm): 17.4- 17.5 (a), 17.7 (j), 46.5 (k), 64.5 (g), 68.6-68.7-69.2 (f), 70.7 (d, e, i, i´), 72.9- 73.5 (c), 75.2- 75.4- 75.6 (b), 121.7 (m),
125.3 (o), 145.5 (p), 152.1 (l), 155.2 (n), 171.5 (h). **MS (MALDI+)**: m/z 5340.8 and 13561.3.

Compound 1-SH. Compound **3** (Scheme S1, 1.7 g, 0.13 mmol) was dissolved in 30 mL of dry dichloromethane (DCM) in a flask. Then, a suspension of cysteamine (50 mg, 0.65 mmol) in 1 mL of DCM was added. The reaction mixture was kept under argon atmosphere at room temperature with stirring overnight. The reaction crude was poured into 200 mL of cold diethyl ether. The precipitate was filtered and washed with cold diethyl ether. The product was obtained as a yellow solid. Yield: 95%.

^{1}H-RMN (500 MHz, CDCl3): δ (ppm): 1.13 (m, 201H, a), 1.23 (s, 6H, j), 2.79-2.95 (m, 8H, n), 3.37-3.76 (m, ~1100H, b, c, d, e, f, m), 4.27 (m, 12H, i, g). **^{13}C-RMN** (125 MHz, CDCl3): δ (ppm): 17.4 (a), 18.3 (j), 38.5 (n), 41.8 (m), 43.5 (k), 64.2 (i), 67.4 (g), 68.7 (f), 70.7 (d, e), 72.9- 73.5 (c), 75.2-75.6 (b), 156.2 (l), 172.9 (h). **MS (MALDI+)**: m/z 5184.5 and 13217.4.

Compound 2-OH. Compound **3** (Scheme S1, 1.6 g, 0.118 mmol) was dissolved in 10 mL dry DCM. A solution of ethanolamine (0.043g, 0.704 mmol) in 2 mL of dry DCM was added. The reaction mixture was stirred for 24 h at room temperature under argon atmosphere. Then, the crude was precipitated into 200 mL of cold diethyl ether and stored in the fridge overnight. The product was filtered and washed with cold diethyl ether to obtain the product as a white powder. Yield: 90 %.

^{1}H-RMN (400 MHz, CDCl3) δ (ppm): 1.12-1.14 (m, 201H, a), 1.21 (s, 6H, j), 3.26-3.30 (m, 8H, m), 3.36-3.74 (m, ~1100H, b, c, d, e, f, n), 4.18-4.30 (m, 12H, i, g), 5.80 (bs, 4H). **^{13}C-RMN** (100 MHz, CDCl3): δ (ppm): 17.3 (a), 17.4 (j), 43. 6 (k), 46.8 (m), 61.7 (n), 65.8 (i) 66.9 (g), 68.9 (f), 70.6 (d, e), 72.9- 73.4 (c), 75.1- 75.5 (b), 156.7 (l), 173.1 (h). **MS (MALDI+)**: m/z 5216 and 13779.

Characterization techniques.

The chemical characterization of compound 1-SH and 2-OH, as well as the intermediates, was carried out by ^{1}H and ^{13}C NMR spectroscopy using a Bruker AV400 spectrometer, and by Matrix-Assisted Laser Desorption/Ionization- Time-Of-Flight (MALDI-TOF) mass spectrometry performed on an Autoflex Bruker mass spectrometer with a dithranol matrix.

The morphology of the NPs, prepared by self-assembly in water of the amphiphilic LDBCs, was studied by TEM. The images were obtained with a FEI TECNAI T20 system with a beam power of 200kV. For sample preparation, a droplet (10 µL) of the freshly prepared sample at the concentration of 1 mg·mL^{-1} in distilled water was deposited on holey carbon film 300 mesh coppered grids provided by Agar Scientific Ltd and an aqueous solution of 3% phosphotungstic acid was used as a negative stain. The grid was dried for 24 hours, prior image acquisition. The average size of the different compounds was calculated by analysing at least 100 structures in 4 TEM images.

Subjects and samples.

Blood samples from four distinct cohorts were procured for experimentation, each pair consisting of appropriate controls for comparison (disease *versus* control groups): patients diagnosed with PDAC and voluntary blood donors (BD); patients with OV and individuals with benign ovarian cysts (OC). Prior to participation in the study, all subjects provided informed consent for inclusion. The study was conducted in compliance with the ethical principles outlined in the Declaration of Helsinki, and the research protocol received approval from the CEICA Ethics Committee (PI22/0133).

There was a sex imbalance between the PDAC (56% male) and control (83% male) cohorts. To assess the potential confounding effect of sex, we performed data quality checks and analyzed intra-group fluorescence signal variability. No significant differences were observed in fluorescence profiles between males and females within each diagnostic group. These results indicate that the observed diagnostic separation is unlikely to be driven by sex-related spectral variation.

Pancreatic cancer (PDAC): Patients (PDAC diseased group) and blood donor subjects (BD control group) cohort sample description.

The control group for this study comprised of blood donor subjects, and both samples and data were sourced from the Blood and Tissue Bank of Aragon and provided by the Biobank of the Aragon Health System, which is a part of the Spanish National Biobanks Network (PT20/00112). Standard operating procedures were followed for processing the samples, and all necessary approvals from the Ethics and Scientific Committees were obtained. The cohort consisted of 100 serum samples collected from Spanish Caucasian individuals who were presumed to be free of any known diseases.

This study also utilized a total of 100 serum samples obtained from patients who received a diagnosis of PDAC at the oncology service of Hospital Universitario Miguel Servet (HUMS) in Zaragoza, Spain. The samples were collected following histopathological confirmation and before the initiation of treatment. They were managed, stored, and provided by the Biobank of the Aragon Health System.

Regarding the distribution of samples based on disease/health status, 50.0% of the samples belonged to the blood donor control group (BD), while 50.0% were obtained from patients diagnosed with pancreatic cancer (PDAC). In terms of sex distribution, out of the total of 200 samples, 139 (69.5%) were from males and 61 (30.5%) were from females. Specifically, within the BD group, 83 out of 100 samples (83.0%) were from males and 17 (17.0%) were from females.

In the PDAC group, 56 out of 100 samples (56.0%) were from males and 44 (44.0%) were from females.

A statistical analysis revealed that, within the BD group, there were no statistically significant differences in age based on gender (p = 0.163, Wilcoxon test). Likewise, within the PDAC group, there were no statistically significant differences in age based on gender (p = 0.647, t-test).

Ovarian Cancer (OV). Ovarian cancer Patients (OV diseased group) and benign cyst patients (OC control group) cohort sample description.

This study included samples obtained from patients diagnosed with ovarian cancer (n=81) and benign cysts (n=81) at the gynecology service of Hospital Virgen del Camino in Pamplona, Spain. The serum samples were collected prior to surgery and were handled, stored, and provided by the Biobank Navarrabiomed, which is part of the Spanish National Biobanks Network (PT17/0015/0007). All procedures followed standard operating protocols and received the necessary approvals from the Ethics and Scientific Committees.

A total of 162 samples were recruited for this study, of which 81 (81/162, 50.0%) were from female patients diagnosed with OV, and 81 (81/162, 50.0%) were from female patients initially suspected to have OV but were ultimately diagnosed with a benign lesion (OC).

Blood sample processing.

Peripheral blood (10 mL) from each subject was collected and transferred into serum separator tubes for subsequent analysis. Tubes were gently mixed immediately after blood collection and then centrifuged at 3200 rpm for 10 min. Separated serum was carefully aspirated to avoid hemolysis and contamination of the separated blood phases, and immediately stored in 1 mL aliquots at -80 °C until analysis.

Fluorescence spectroscopy.

Serum samples obtained from the four cohorts of patients were diluted 1:25 in phosphate buffered saline (PBS). Experiments involving nanoparticles were conducted at a final concentration of 500 µg·mL-1 prior to data acquisition. A total volume of 50 µL was transferred into non-skirted 96-well microplates (4titude, UK). Samples were incubated for 30 minutes to allow the interaction of the fluorescent probes with the albumin present in each sample and fluorescence measurements were performed using a CLARIOstar plate reader (BMG Labtech, Germany) with an excitation wavelength of 330 and 350 nm, using excitation and emission bandwidths of 10 nm. Fluorescence emission spectra were recorded ranging from 400 to 700 nm. To assess signal stability, a set of 3 serum samples randomly selected from healthy controls and one commercial fetal bovine serum (FBS) sample were subjected to three independent freeze–thaw cycles. The resulting emission spectra showed high reproducibility, with mean Pearson correlation coefficients exceeding 0.997 at 330 nm and 0.9985 at 350 nm across all samples, confirming that the freeze–thaw process did not affect the fluorescence profiles (Figure S1).

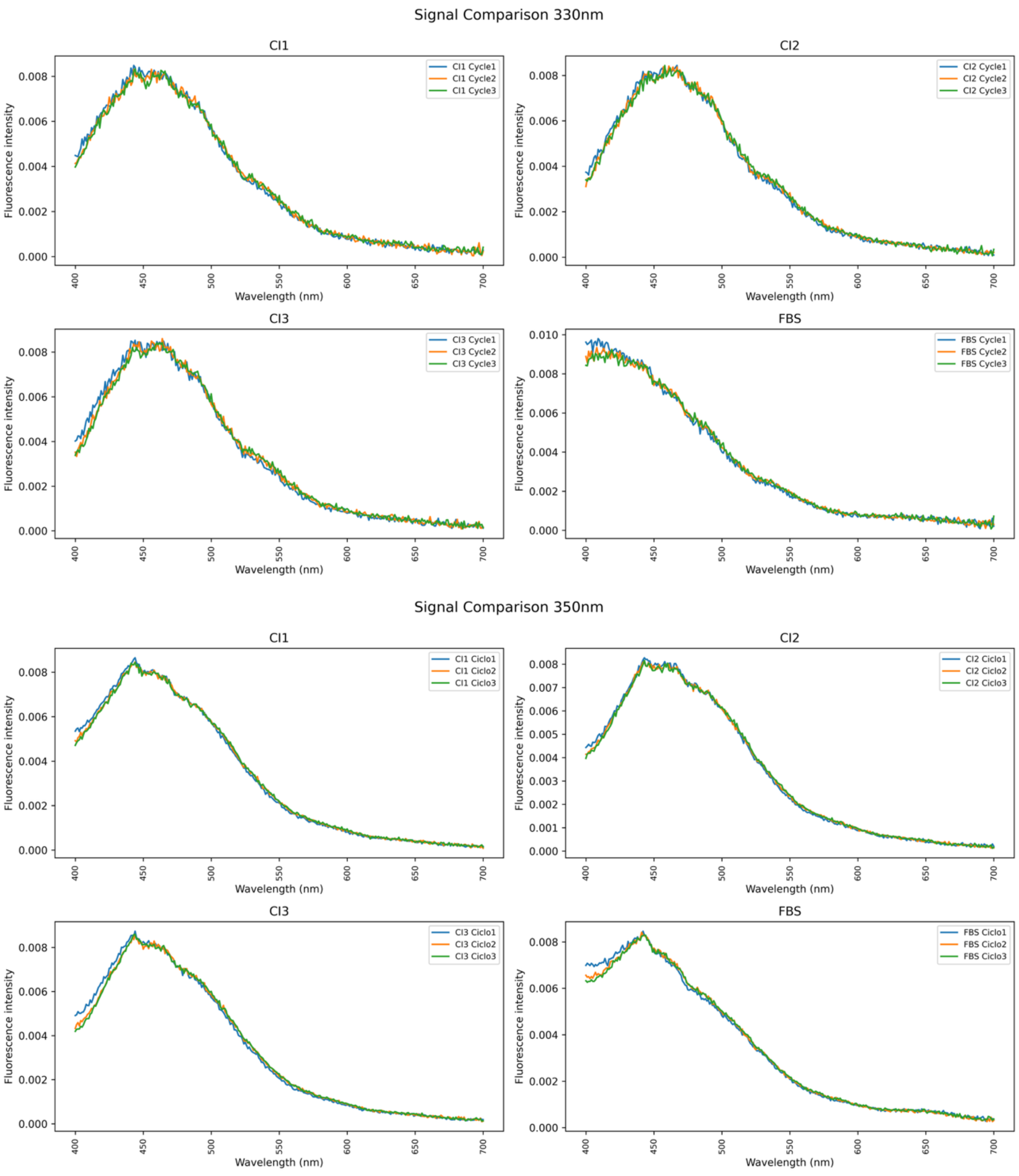

Figure S1. Fluorescence emission spectra of four biological samples 3 controls (CI1, CI2, CI3) and commercial fetal bovine serum (FBS) recorded after three consecutive freeze–thaw cycles. Each subplot corresponds to a different sample, and each line represents one cycle. Spectra were normalized to unit area and measured at two excitation wavelengths: (A) 330 nm and (B) 350 nm. The high overlap between replicates demonstrates excellent signal stability. Mean Pearson correlation coefficients across cycles were >0.997 at 330 nm and >0.9985 at 350 nm for all samples.

Machine Learning Algorithms.

Selection of Algorithms. *Rationale for Choice*. The selection of machine learning algorithms for this study was guided by their proven efficacy in handling complex, high-dimensional datasets typical in fluorescence spectroscopy. Given the intricate nature of spectral data, it was imperative to employ algorithms capable of effectively parsing these nuanced datasets. Simpler algorithms were also used so that all they can be compared. Gaussian Naive Bayes, logistic regression and K-Nearest Neighbors (KNN) were chosen for their simplicity. Ensemble methods, such as AdaBoost, CatBoost and XGBoost, were chosen for their robustness in managing datasets with complex underlying structures and their ability to improve prediction accuracy by combining multiple weak learners into a strong model. These methods are particularly adept at handling the subtle variations and high dimensionality inherent in fluorescence spectral data, making them well-suited for this study.

***List of Algorithms*.** The machine learning algorithms selected for this study were chosen based on their ability to address the specific characteristics of the data and the task. These algorithms were categorized into six groups: ensemble methods, tree-based methods, probabilistic methods, distance-based methods, support vector machines (SVM), and linear models. Ensemble techniques, such as AdaBoost, CatBoost, and XGBoost, were implemented due to their effectiveness in improving predictive accuracy and reducing overfitting. AdaBoost enhances the performance of weak classifiers through sequential training and misclassification weight adjustment,[1] while CatBoost was chosen for its optimization in handling categorical features and its reduced need for extensive preprocessing.[2] XGBoost was selected for its scalability and ability to prevent overfitting through regularization.[3] Tree-based methods, including Decision Trees and Random Forest, were selected for their interpretability and robustness. Decision Trees, although prone to overfitting, are easy to visualize and understand,[4] while Random Forest reduces variance and overfitting by combining multiple decision trees trained on different data subsets.[5]

Probabilistic methods, such as Gaussian Naive Bayes, were included for their computational efficiency and theoretical foundations in probability, despite assuming independence between features.[6] The KNN algorithm was chosen from distance-based methods due to its simplicity and interpretability, although it becomes computationally expensive with larger datasets.[7] SVM were selected for their capability to handle both linear and non-linear classification tasks, particularly in high-dimensional spaces with complex decision boundaries.[8] Lastly, Logistic Regression, a linear model, was used as a baseline for binary classification due to its simplicity

---

[1] Freund, Y., & Schapire, R. E. A decision-theoretic generalization of on-line learning and an application to boosting. *J. Comput. Syst. Sci.*, **1997**, *55*(1), 119-139.

[2] Prokhorenkova, L., Gusev, G., Vorobev, A., Dorogush, A. V.; Gulin, A. CatBoost: unbiased boosting with categorical features. Advances in Neural Information Processing Systems, **2018**, 31. https://proceedings.neurips.cc/paper/2018/file/14491b756b3a51daac41c24863285549-Paper.pdf

[3] Chen, T.; Guestrin, C. XGBoost: A scalable tree boosting system. Proceedings of the 22nd ACM SIGKDD International Conference on Knowledge Discovery and Data Mining 785-794. **2016**. ACM.

[4] Breiman, L., Friedman, J., Olshen, R. A., & Stone, C. J. Classification and Regression Trees. Wadsworth International Group. 1984. ISBN: 978-0412048418

[5] Breiman, L. Random Forests. *Machine Learning*, **2001**, *45*, 5-32.

[6] Hastie, T.; Tibshirani, R.; Friedman, J. The Elements of Statistical Learning: Data Mining, Inference, and Prediction (2nd ed.). Springer. 2009. ISBN: 978-0387848570

[7] Cover, T.; Hart, P. "Nearest neighbor pattern classification." *IEEE Transactions on Information Theory*, **1967**, *13*, 21-27.

[8] Cortes, C.; Vapnik, V. Support-vector networks. Machine Learning, **1995**, 20, 273-297.

and effectiveness in linearly separable data[9] and further supported by practical approaches from the work of Hosmer D. W. et al. [10]

All algorithms were implemented using the scikit-learn library (version 1.02), with hyperparameter tuning performed using grid search and cross-validation. For ensemble methods such as XGBoost and CatBoost, parameters such as learning rate, number of estimators, and tree depth were optimized. In the case of SVM, kernel type and regularization parameters were fine-tuned to ensure proper model fitting. The K value for KNN was selected based on minimizing the validation error. Model performance was evaluated using a 5-fold cross-validation technique to ensure robust performance estimation. All experiments were conducted using a consistent computational environment to ensure reliable results.

Data Preprocessing. *Spectral Data Preparation*. The fluorescence spectral data underwent several preprocessing steps to ensure optimal analysis conditions. The normalization technique was not applied to the samples since they come from the subtraction of the data obtained from the fluorescence analysis applied to the same biological sample, but with or without the addition of nanoparticles.

A Savitzky-Golay filter was then applied since the values included a very high level of noise that did not provide any information. This noise is common in fluorescence measurements.

In addition, to improve the performance of the algorithms and minimize the number of parameters, the samples were decimated, reducing the number of data points per sample (from 300 to 60), since no information was lost with this reduction.

Training and Test Sets. The dataset was divided into training and testing sets, adhering to a standard ratio (commonly 70:30) to ensure both adequate training of the models and robust validation of their predictive capabilities. Stratification methods were employed to maintain consistent distribution of classes in both sets, crucial for maintaining the integrity of the model evaluation, especially in cases of class imbalance.

The K-fold method (with K = 10) was used to evaluate the methods. All samples belonged to a test group in one of the 10 iterations with a 90-10 split for training and testing.

In other words, K trainings were performed with each algorithm, using K-1 groups of samples for training and 1 for testing. In each iteration, the test group is different, and, over the K iterations, it covers the total of the input samples.

These predictions made on the test data were stored so that the total group of samples generated as many results.

This technique is highly recommended for situations with a limited number of samples, as is the case study, and allows for a very good evaluation of the method.

The results presented correspond to the statistics obtained from the application of the K-fold method on the samples, with each of the algorithms.

This evaluation of the method covers the type and architecture of the classification model used.

If this method is used for new samples, the mean of the estimates of the K models could be applied, or a new model could be trained with a specific split, with the confidence that the model offers performance obtained in the method evaluation stage.

Parameter Tuning and Model Training. *Hyperparameter Optimization*. Each algorithm underwent a hyperparameter tuning process. Several sets of hyperparameters were employed to explore a range of parameter combinations, identifying the set that maximized model performance.

Training Process. The models were trained over numerous iterations, with careful monitoring of learning rates (where applicable) to optimize the convergence process. The K-fold cross-

[9] Cox, D. R. The regression analysis of binary sequences. *Journal of the Royal Statistical Society: Series B* (Methodological), **1958**, *20*, 215-242.
[10] Hosmer, D. W.; Lemeshow, S.; Sturdivant, R. X. Applied Logistic Regression (3rd ed.). Wiley. 2013. ISBN: 978-0470582473

validation technique was used for the training process, which is highly appropriate for situations with a limited number of samples.

Model Evaluation and Validation. *Evaluation Metrics*. The performance of the models was evaluated using several key metrics, including the ROC-AUC, accuracy, precision, recall, and F1 score. These metrics were chosen to provide a comprehensive view of each model's performance, particularly in handling the imbalanced nature of the spectral data. AUC was used to assess the model's ability to distinguish between classes independently of the classification threshold, while accuracy provided an overall measure of correct classifications. Precision and recall were used to examine the balance between false positives and false negatives, which is crucial in imbalanced datasets. The F1 score, as the harmonic mean of precision and recall, was also reported to give a single measure of model performance in scenarios where neither precision nor recall should be prioritized.

Cross-Validation. In addition to these metrics, K-fold cross-validation was employed to validate the models. In this study, 10-fold cross-validation was used, which involves dividing the dataset into 10 equal subsets. Each subset is used once as the validation set while the model is trained on the remaining 9 subsets. This process was repeated until every subset has been used as the validation set. By averaging the results across all folds, this technique provides a more reliable estimate of model performance, helping to mitigate issues such as overfitting and ensuring that the performance metrics are representative of the overall data distribution.

Algorithm-Specific Considerations. Each algorithm had unique considerations, and specific set of hyperparameters were selected to each algorithm.

Software and Computational Resources. *Software Used*. The analysis utilized advanced software and programming languages, predominantly Python, known for its extensive machine learning libraries like scikit-learn. *Computational Resources*. The computational demands of this study were met using high-performance CPUs and GPUs, ensuring efficient processing of the complex machine learning tasks.

330nm	Algorithm	1-SH	2-OH	3-NH_3^+
PDAC	XGBoost	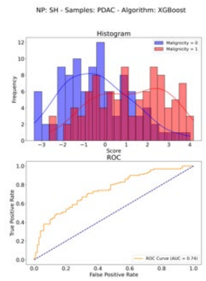	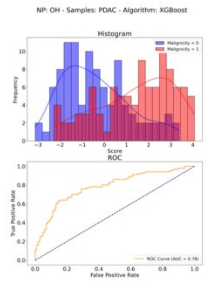	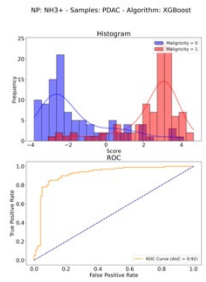
	AdaBoost	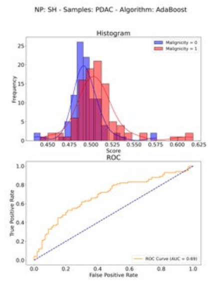	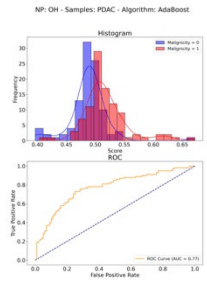	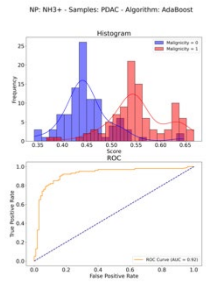
	CatBoost	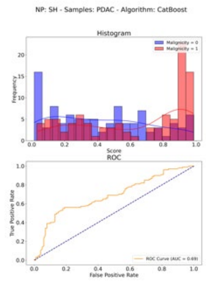	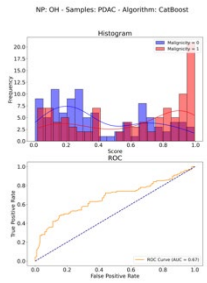	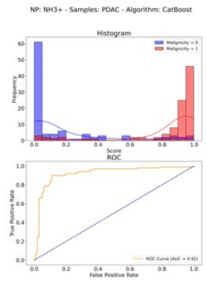
OV	RandomForest	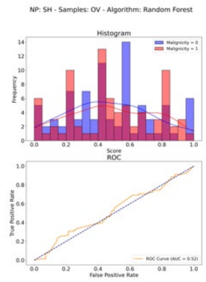	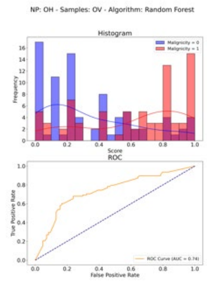	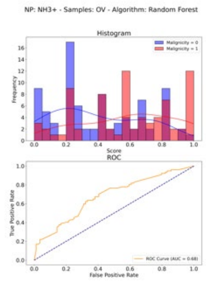
	AdaBoost	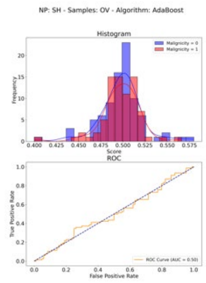	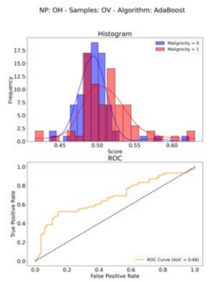	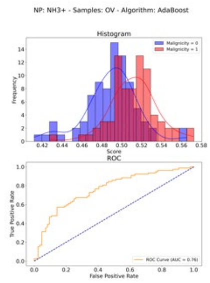

330nm	Algorithm	1-SH	2-OH	3-NH$_3^+$
	LogisticRegression	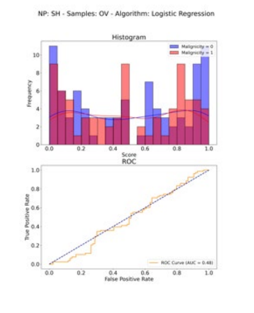	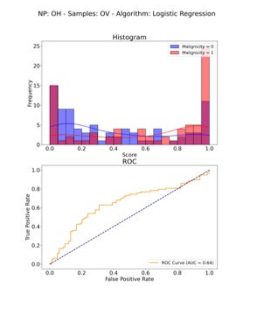	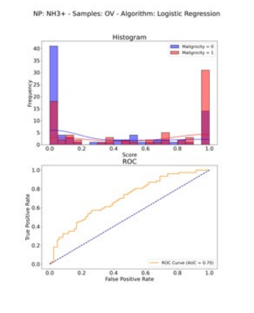

350nm	Algorithm	1-SH	2-OH	3-NH$_3^+$
PDAC	KNeighborsClassifier	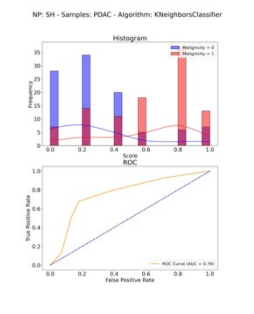	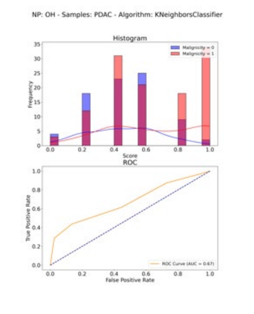	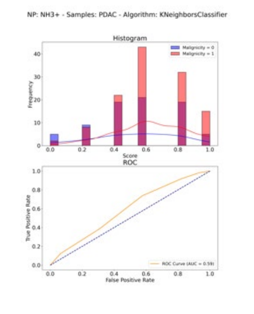
	Logistic Regression	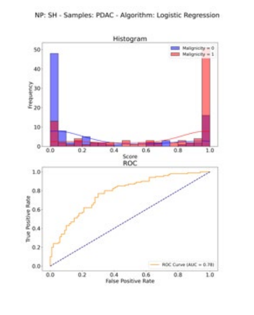	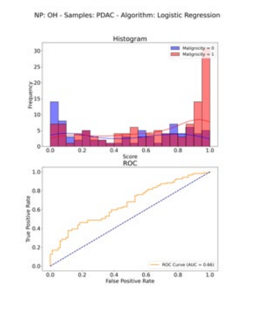	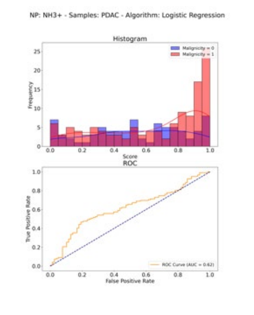
	XGBoost	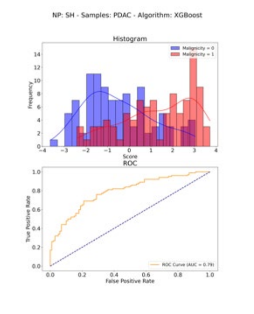	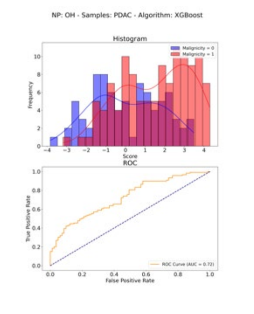	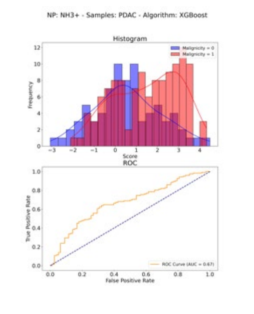
OV	Logistic Regression	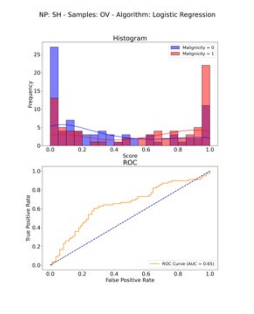	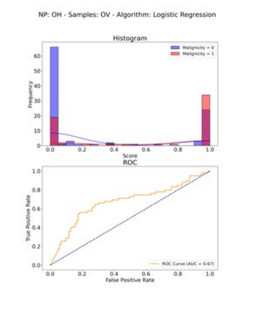	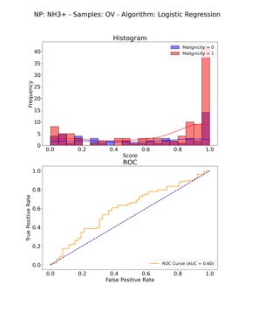

	CatBoost	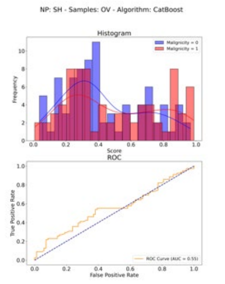	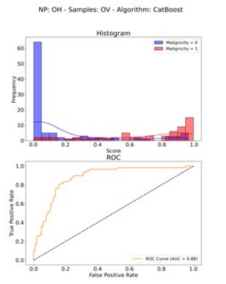	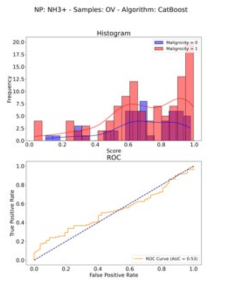
	Random Forest	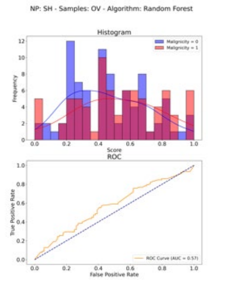	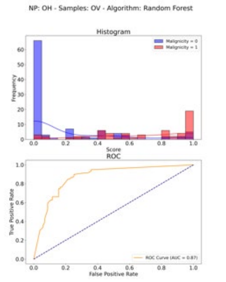	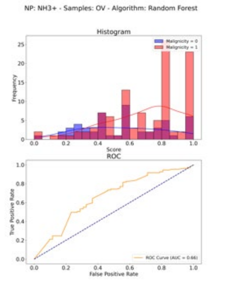

Figure S2: Comparative ROC Curve Analysis and Probability Distribution Histograms for NP-Assisted Fluorescence Spectroscopy in Pathology Detection. The top panel displays results for 330 nm excitation wavelength, and the bottom panel for 350 nm. Each subplot corresponds to the combination of a specific machine learning algorithm and nanoparticle (NP) type. ROC curves are plotted in the insets, with true positive rates (sensitivity) on the y-axis against false positive rates (1-specificity) on the x-axis. The AUC values are indicative of the model's ability to discriminate between the pathological and non-pathological states. The histograms exhibit the distribution of predicted probabilities for pathological (red bars) and non-pathological (blue bars) conditions, with a clear separation suggesting a higher model performance. NP sample Serum was diluted 1:25 in PBS, with NP concentration of 500 µg mL^{-1}, excitation wavelength 330 nm or 350 nm, and emission spectra from 400 to 700 nm.

Table S1. Different evaluation values (global mean value and the standard deviation in parentheses for each evaluation metric), for the different algorithms applied to PDAC samples excited either at 330nm and 350nm. Gray background indicates the cell with highest value for that evaluation metrics and excitation wavelength, and bold text indicates the highest value for the specific evaluation metric, algorithm and nanoparticle.

330nm	Model	accuracy	precision	recall	specificity	f1
1-SH_PDAC	AdaBoost	*0.640 (0.080)*	*0.663 (0.105)*	*0.570 (0.127)*	*0.710 (0.082)*	*0.613 (0.109)*
	CatBoost	*0.605 (0.091)*	*0.600 (0.110)*	*0.630 (0.155)*	*0.580 (0.127)*	*0.615 (0.112)*
	XGBoost	***0.680** (0.078)*	*0.700 (0.124)*	***0.630** (0.098)*	*0.730 (0.114)*	*0.663 (0.088)*
	DecissionTree	*0.640 (0.080)*	*0.656 (0.120)*	*0.590 (0.067)*	*0.690 (0.169)*	*0.621 (0.053)*
	Random Forest	*0.655 (0.101)*	*0.663 (0.119)*	*0.630 (0.121)*	*0.680 (0.140)*	*0.646 (0.103)*
	GaussianNB	*0.670 (0.075)*	*0.793 (0.116)*	*0.460 (0.152)*	*0.880 (0.076)*	*0.582 (0.130)*
	KNeighborsClassifier	*0.660 (0.104)*	*0.678 (0.154)*	*0.610 (0.137)*	*0.710 (0.176)*	*0.642 (0.105)*
	SVM	*0.590 (0.062)*	***0.821** (0.168)*	*0.230 (0.104)*	***0.950** (0.050)*	*0.359 (0.121)*
	Logistic Regression	*0.670 (0.108)*	*0.673 (0.116)*	*0.660 (0.162)*	*0.680 (0.135)*	***0.667** (0.124)*
2-OH_PDAC	AdaBoost	***0.735** (0.118)*	*0.737 (0.149)*	***0.730** (0.143)*	*0.740 (0.189)*	***0.734** (0.113)*
	CatBoost	*0.635 (0.087)*	*0.636 (0.099)*	*0.630 (0.113)*	*0.640 (0.092)*	*0.633 (0.094)*
	XGBoost	*0.715 (0.121)*	*0.722 (0.146)*	*0.700 (0.146)*	*0.730 (0.158)*	*0.711 (0.129)*
	DecissionTree	*0.695 (0.104)*	*0.689 (0.112)*	*0.710 (0.175)*	*0.680 (0.173)*	*0.700 (0.116)*
	Random Forest	*0.700 (0.122)*	*0.696 (0.157)*	*0.710 (0.116)*	*0.690 (0.234)*	*0.703 (0.090)*
	GaussianNB	*0.635 (0.121)*	*0.690 (0.177)*	*0.490 (0.187)*	*0.780 (0.170)*	*0.573 (0.164)*

	KNeighborsClassifier	0.720 (0.108)	0.724 (0.138)	0.710 (0.137)	0.730 (0.167)	0.717 (0.099)
	SVM	0.630 (0.090)	**0.825** (0.161)	0.330 (0.145)	**0.930** (0.091)	0.471 (0.163)
	Logistic Regression	0.620 (0.103)	0.622 (0.110)	0.610 (0.170)	0.630 (0.135)	0.616 (0.125)
3-NH3+_PDAC	AdaBoost	0.850 (0.074)	0.850 (0.090)	0.850 (0.077)	0.850 (0.107)	0.850 (0.072)
	CatBoost	**0.880** (0.051)	**0.865** (0.078)	**0.900** (0.074)	**0.860** (0.099)	**0.882** (0.050)
	XGBoost	0.855 (0.085)	0.845 (0.112)	0.870 (0.090)	0.840 (0.144)	0.857 (0.080)
	DecissionTree	0.825 (0.087)	0.828 (0.108)	0.820 (0.114)	0.830 (0.110)	0.824 (0.091)
	Random Forest	0.855 (0.079)	0.845 (0.106)	0.870 (0.092)	0.840 (0.148)	0.857 (0.074)
	GaussianNB	0.825 (0.129)	0.828 (0.147)	0.820 (0.124)	0.830 (0.170)	0.824 (0.126)
	KNeighborsClassifier	0.855 (0.069)	0.838 (0.103)	0.880 (0.071)	0.830 (0.123)	0.859 (0.065)
	SVM	0.830 (0.056)	0.837 (0.091)	0.820 (0.081)	0.840 (0.107)	0.828 (0.050)
	Logistic Regression	0.675 (0.084)	0.673 (0.095)	0.680 (0.131)	0.670 (0.111)	0.677 (0.099)

350nm	Model	accuracy	precision	recall	specificity	f1
1-SH_PDAC	AdaBoost	0.720 (0.103)	0.744 (0.134)	0.670 (0.137)	0.770 (0.141)	0.705 (0.110)
	CatBoost	0.675 (0.072)	0.677 (0.093)	0.670 (0.106)	0.680 (0.106)	0.673 (0.076)
	XGBoost	0.720 (0.119)	0.734 (0.130)	**0.690** (0.123)	0.750 (0.142)	0.711 (0.115)
	DecissionTree	0.665 (0.136)	0.670 (0.148)	0.650 (0.150)	0.680 (0.174)	0.660 (0.138)
	Random Forest	0.655 (0.099)	0.674 (0.161)	0.600 (0.162)	0.710 (0.190)	0.635 (0.106)

	GaussianNB	*0.685 (0.063)*	*0.785 (0.125)*	*0.510 (0.103)*	*0.860 (0.081)*	*0.618 (0.096)*
	KNeighborsClassifier	***0.750*** *(0.087)*	*0.791 (0.090)*	*0.680 (0.130)*	*0.820 (0.083)*	***0.731*** *(0.096)*
	SVM	*0.600 (0.071)*	***0.857*** *(0.117)*	*0.240 (0.121)*	***0.960*** *(0.048)*	*0.375 (0.144)*
	Logistic Regression	*0.710 (0.077)*	*0.714 (0.100)*	*0.700 (0.140)*	*0.720 (0.127)*	*0.707 (0.077)*
2-OH_PDAC	AdaBoost	*0.650 (0.084)*	*0.713 (0.143)*	*0.689 (0.144)*	*0.593 (0.206)*	*0.701 (0.110)*
	CatBoost	***0.665*** *(0.074)*	*0.717 (0.098)*	*0.723 (0.096)*	*0.580 (0.161)*	*0.720 (0.084)*
	XGBoost	*0.620 (0.056)*	*0.687 (0.114)*	*0.664 (0.090)*	*0.556 (0.126)*	*0.675 (0.060)*
	DecissionTree	*0.635 (0.063)*	*0.709 (0.107)*	*0.655 (0.088)*	*0.605 (0.188)*	*0.681 (0.069)*
	Random Forest	*0.640 (0.083)*	*0.697 (0.127)*	*0.697 (0.113)*	*0.556 (0.199)*	*0.697 (0.077)*
	GaussianNB	*0.640 (0.092)*	***0.841*** *(0.119)*	*0.487 (0.117)*	***0.864*** *(0.129)*	*0.617 (0.107)*
	KNeighborsClassifier	*0.590 (0.104)*	*0.670 (0.134)*	*0.613 (0.100)*	*0.556 (0.182)*	*0.640 (0.093)*
	SVM	*0.600 (0.136)*	*0.600 (0.128)*	***0.983*** *(0.044)*	*0.037 (0.084)*	***0.745*** *(0.105)*
	Logistic Regression	*0.610 (0.092)*	*0.669 (0.118)*	*0.681 (0.142)*	*0.506 (0.133)*	*0.675 (0.110)*
3-NH3+_PDAC	AdaBoost	*0.650 (0.124)*	*0.703 (0.123)*	*0.738 (0.143)*	*0.513 (0.138)*	*0.720 (0.113)*
	CatBoost	*0.630 (0.105)*	*0.671 (0.126)*	*0.770 (0.104)*	*0.410 (0.137)*	*0.718 (0.105)*
	XGBoost	*0.615 (0.092)*	*0.683 (0.111)*	*0.689 (0.089)*	*0.500 (0.192)*	*0.686 (0.090)*
	DecissionTree	*0.625 (0.098)*	*0.679 (0.099)*	*0.730 (0.136)*	*0.462 (0.206)*	*0.704 (0.089)*
	Random Forest	***0.660*** *(0.118)*	***0.718*** *(0.116)*	*0.730 (0.149)*	*0.551 (0.167)*	*0.724 (0.109)*

GaussianNB	*0.635 (0.100)*	*0.713 (0.093)*	*0.672 (0.101)*	***0.577*** *(0.157)*	*0.692 (0.070)*
KNeighborsClassifier	*0.615 (0.067)*	*0.667 (0.105)*	*0.738 (0.115)*	*0.423 (0.114)*	*0.700 (0.086)*
SVM	*0.605 (0.111)*	*0.608 (0.112)*	***0.992*** *(0.021)*	*0.000 (0.000)*	***0.754*** *(0.087)*
Logistic Regression	*0.590 (0.058)*	*0.656 (0.114)*	*0.689 (0.079)*	*0.436 (0.127)*	*0.672 (0.069)*

Table S2. Different evaluation values (global mean value and the standard deviation in parentheses for each evaluation metric), for the different algorithms applied to OV samples excited either at 330nm and 350nm. Gray background indicates the cell with highest value for that evaluation metrics and excitation wavelength, and bold text indicates the highest value for the specific evaluation metric, algorithm and nanoparticle.

330nm	Model	accuracy	precision	recall	specificity	f1
1-SH_OV	AdaBoost	*0.475 (0.089)*	*0.455 (0.136)*	*0.449 (0.124)*	*0.500 (0.124)*	*0.452 (0.101)*
	CatBoost	*0.481 (0.096)*	*0.463 (0.197)*	*0.487 (0.164)*	*0.476 (0.200)*	*0.475 (0.116)*
	XGBoost	***0.568*** *(0.100)*	***0.549*** *(0.172)*	*0.577 (0.161)*	***0.560*** *(0.164)*	*0.563 (0.112)*
	DecissionTree	*0.531 (0.096)*	*0.512 (0.175)*	*0.538 (0.146)*	*0.524 (0.138)*	*0.525 (0.132)*
	Random Forest	*0.494 (0.111)*	*0.471 (0.187)*	*0.423 (0.155)*	*0.560 (0.175)*	*0.446 (0.128)*
	GaussianNB	*0.543 (0.116)*	*0.520 (0.124)*	***0.667*** *(0.201)*	*0.429 (0.225)*	***0.584*** *(0.130)*
	KNeighborsClassifier	*0.556 (0.103)*	*0.537 (0.106)*	*0.564 (0.081)*	*0.548 (0.158)*	*0.550 (0.080)*
	SVM	*0.451 (0.119)*	*0.450 (0.178)*	*0.628 (0.294)*	*0.286 (0.277)*	*0.524 (0.190)*
	Logistic Regression	*0.475 (0.073)*	*0.455 (0.226)*	*0.449 (0.208)*	*0.500 (0.226)*	*0.452 (0.131)*
2-OH_OV	AdaBoost	*0.611 (0.082)*	*0.600 (0.198)*	*0.577 (0.112)*	*0.643 (0.190)*	*0.588 (0.066)*
	CatBoost	*0.660 (0.134)*	*0.658 (0.145)*	*0.615 (0.248)*	*0.702 (0.141)*	*0.636 (0.149)*
	XGBoost	*0.648 (0.049)*	*0.644 (0.155)*	*0.603 (0.175)*	*0.690 (0.138)*	*0.623 (0.085)*
	DecissionTree	*0.654 (0.064)*	*0.628 (0.129)*	***0.692*** *(0.136)*	*0.619 (0.159)*	*0.659 (0.061)*
	Random Forest	*0.698 (0.116)*	*0.688 (0.110)*	*0.679 (0.184)*	*0.714 (0.129)*	***0.684*** *(0.117)*
	GaussianNB	***0.728*** *(0.115)*	***0.783*** *(0.171)*	*0.603 (0.178)*	***0.845*** *(0.149)*	*0.681 (0.141)*

	KNeighborsClassifier	*0.691 (0.115)*	*0.706 (0.128)*	*0.615 (0.211)*	*0.762 (0.132)*	*0.658 (0.112)*
	SVM	*0.562 (0.155)*	*0.539 (0.244)*	*0.615 (0.337)*	*0.512 (0.278)*	*0.575 (0.246)*
	Logistic Regression	*0.642 (0.181)*	*0.628 (0.192)*	*0.628 (0.219)*	*0.655 (0.247)*	*0.628 (0.183)*
3-NH3+_OV	AdaBoost	***0.677*** *(0.073)*	*0.654 (0.082)*	*0.688 (0.217)*	*0.667 (0.114)*	***0.671*** *(0.120)*
	CatBoost	*0.652 (0.091)*	*0.627 (0.133)*	*0.675 (0.175)*	*0.631 (0.191)*	*0.650 (0.131)*
	XGBoost	*0.677 (0.128)*	***0.667*** *(0.177)*	*0.649 (0.202)*	*0.702 (0.163)*	*0.658 (0.130)*
	DecissionTree	*0.584 (0.147)*	*0.569 (0.237)*	*0.532 (0.191)*	*0.631 (0.197)*	*0.550 (0.182)*
	Random Forest	*0.646 (0.178)*	*0.628 (0.226)*	*0.636 (0.221)*	*0.655 (0.183)*	*0.632 (0.205)*
	GaussianNB	*0.491 (0.117)*	*0.478 (0.161)*	***0.714*** *(0.219)*	*0.286 (0.121)*	*0.573 (0.156)*
	KNeighborsClassifier	*0.627 (0.114)*	*0.608 (0.213)*	*0.623 (0.170)*	*0.631 (0.229)*	*0.615 (0.105)*
	SVM	*0.540 (0.131)*	*0.537 (0.319)*	*0.286 (0.167)*	***0.774*** *(0.221)*	*0.373 (0.169)*
	Logistic Regression	*0.627 (0.154)*	*0.616 (0.210)*	*0.584 (0.229)*	*0.667 (0.176)*	*0.600 (0.185)*

350nm	Model	accuracy	precision	recall	specificity	f1
1-SH_OV	AdaBoost	*0.599 (0.107)*	*0.587 (0.121)*	*0.564 (0.164)*	*0.631 (0.134)*	*0.575 (0.107)*
	CatBoost	*0.568 (0.105)*	*0.563 (0.139)*	*0.462 (0.190)*	*0.667 (0.120)*	*0.507 (0.143)*
	XGBoost	*0.599 (0.157)*	*0.592 (0.174)*	*0.538 (0.208)*	*0.655 (0.126)*	*0.564 (0.181)*
	DecissionTree	*0.574 (0.115)*	*0.553 (0.142)*	***0.603*** *(0.151)*	*0.548 (0.156)*	*0.577 (0.126)*
	Random Forest	*0.549 (0.076)*	*0.535 (0.150)*	*0.487 (0.102)*	*0.607 (0.146)*	*0.510 (0.073)*

	GaussianNB	*0.512 (0.074)*	*0.492 (0.163)*	*0.385 (0.102)*	*0.631 (0.137)*	*0.432 (0.094)*
	KNeighborsClassifier	*0.494 (0.148)*	*0.473 (0.143)*	*0.449 (0.159)*	*0.536 (0.159)*	*0.461 (0.148)*
	SVM	*0.481 (0.104)*	*0.313 (0.100)*	*0.064 (0.300)*	***0.869*** *(0.271)*	*0.106 (0.150)*
	Logistic Regression	***0.654*** *(0.115)*	***0.653*** *(0.199)*	*0.603 (0.173)*	*0.702 (0.175)*	***0.627*** *(0.135)*
2-OH_OV	AdaBoost	***0.858*** *(0.096)*	***0.810*** *(0.142)*	*0.797 (0.153)*	*0.893 (0.105)*	*0.803 (0.113)*
	CatBoost	*0.821 (0.095)*	*0.750 (0.215)*	*0.763 (0.157)*	*0.854 (0.116)*	*0.756 (0.161)*
	XGBoost	*0.833 (0.088)*	*0.776 (0.202)*	*0.763 (0.130)*	*0.874 (0.098)*	*0.769 (0.157)*
	DecissionTree	*0.827 (0.113)*	*0.754 (0.224)*	*0.780 (0.149)*	*0.854 (0.138)*	*0.767 (0.173)*
	Random Forest	*0.778 (0.082)*	*0.709 (0.227)*	*0.661 (0.151)*	*0.845 (0.130)*	*0.684 (0.144)*
	GaussianNB	*0.741 (0.078)*	*0.660 (0.108)*	*0.593 (0.234)*	*0.825 (0.063)*	*0.625 (0.123)*
	KNeighborsClassifier	*0.852 (0.079)*	*0.761 (0.163)*	***0.864*** *(0.092)*	*0.845 (0.095)*	***0.810*** *(0.114)*
	SVM	*0.617 (0.080)*	*0.000 (0.000)*	*0.000 (0.000)*	***0.971*** *(0.039)*	*0.000 (0.000)*
	Logistic Regression	*0.685 (0.119)*	*0.563 (0.247)*	*0.610 (0.260)*	*0.728 (0.155)*	*0.585 (0.233)*
3-NH3+_OV	AdaBoost	*0.590 (0.090)*	*0.701 (0.111)*	*0.688 (0.183)*	*0.385 (0.265)*	*0.694 (0.123)*
	CatBoost	*0.621 (0.069)*	*0.676 (0.089)*	*0.844 (0.094)*	*0.154 (0.133)*	*0.751 (0.062)*
	XGBoost	*0.658 (0.107)*	*0.725 (0.124)*	*0.798 (0.106)*	*0.365 (0.245)*	*0.760 (0.094)*
	DecissionTree	*0.596 (0.125)*	*0.712 (0.080)*	*0.679 (0.196)*	***0.423*** *(0.207)*	*0.695 (0.116)*
	Random Forest	*0.665 (0.117)*	***0.743*** *(0.146)*	*0.771 (0.125)*	*0.442 (0.319)*	*0.757 (0.101)*

GaussianNB	*0.615* *(0.097)*	*0.708* *(0.122)*	*0.734* *(0.140)*	*0.365* *(0.165)*	*0.721* *(0.078)*
KNeighborsClassifier	*0.627* *(0.105)*	*0.679* *(0.114)*	*0.853* *(0.114)*	*0.154* *(0.151)*	*0.756* *(0.081)*
SVM	***0.671*** *(0.104)*	*0.675* *(0.109)*	***0.991*** *(0.023)*	*0.000* *(0.000)*	***0.803*** *(0.080)*
Logistic Regression	*0.627* *(0.075)*	*0.725* *(0.131)*	*0.725* *(0.112)*	*0.423* *(0.234)*	*0.725* *(0.067)*
